# A Framework for Characterization of Optimal Decision Rules in Hypothesis-Testing Problems

**DOI:** 10.3390/e28060657

**Published:** 2026-06-09

**Authors:** Emre Efendi, Berkan Dulek, Sinan Gezici, Yanglei Song

**Affiliations:** 1 Department of Electrical and Electronics Engineering, Hacettepe University, Beytepe Campus, Ankara 06800, Turkey; eefendi@ee.hacettepe.edu.tr (E.E.); berkan@ee.hacettepe.edu.tr (B.D.); 2 Department of Electrical and Electronics Engineering, Bilkent University, Ankara 06800, Turkey; 3 Department of Mathematics and Statistics, Queen’s University, Kingston, ON K7L 3N6, Canada; yanglei.song@queensu.ca

**Keywords:** decision rule, hypothesis testing, Bayes risk, Neyman–Pearson criterion, prospect theory

## Abstract

In this review paper, we present a framework for the characterization of optimal decision rules in *M*-ary hypothesis-testing problems where the performance metric is defined as a function of pairwise error probabilities. This framework is based on the approaches developed in several recent studies in the literature, which are unified and presented in a tutorial fashion in this paper. A pairwise error probability represents the probability of selecting a specific hypothesis when a different hypothesis is true, and can be stacked into a pairwise probability vector for a given problem. In the considered framework, instead of optimizing the performance metric of interest over the infinite-dimensional set of all possible decision rules, the optimization is performed directly over the compact and convex set of all achievable pairwise probability vectors. We demonstrate that any pairwise probability vector within this feasible set can be realized via a randomization of at most two likelihood ratio quantizers (LRQs) with different sets of parameters. While one of these LRQs can always be selected as a deterministic LRQ, the other one is possibly a randomized LRQ, which can be written as a randomization of at most M(M−1) deterministic LRQs, with *M* denoting the number of hypotheses. The main advantage of this framework is that it allows for the attainment of pairwise probability vectors that do not reside on the boundary of the feasible set and that are fundamentally inaccessible via LRQs, which are optimal for classical performance metrics such as the Bayes risk or the Neyman–Pearson criterion. Furthermore, we show that the characterization of decision rules with the presented framework is particularly advantageous for performance metrics based on prospect theory (PT), such as behavioral utility. Specifically, it is demonstrated that the optimal pairwise probability vector for a PT-based metric is not guaranteed to lie on the boundary of the feasible set of pairwise probability vectors. This results in suboptimal performance achieved by LRQs for such performance metrics. On the other hand, the randomized decision rules characterized in this paper can achieve pairwise probability vectors located in the interior of the feasible set, thereby yielding optimal performance. Numerical results corroborate these findings, demonstrating that the decision rules characterized within our framework yield optimal behavioral utility-based performance scores.

## 1. Introduction

In his seminal book [1], H. Vincent Poor characterizes detection and estimation theory as the study of drawing inferences about a phenomenon of interest (POI) from noisy observations. This paper focuses primarily on detection problems (also called hypothesis-testing problems), which involve identifying the true underlying state of the POI. In such problems, observations whose probability distributions depend on the true state of the POI are processed by a decision rule, or detector. Traditional performance metrics frequently employed to evaluate such decision rules include the *Bayes risk*, the *Neyman–Pearson (NP) criterion*, and the *minimax criterion*. As demonstrated in [1,2,3], likelihood ratio tests (LRTs) are proven to be optimal for these classical performance metrics. These classical metrics rely on the assumption that the decision agent (DA) is rational, much like physical sensors, which function as rigid, objective measurement devices.

Classical performance metrics may be inadequate in scenarios where human operators are integrated into detection systems, as human judgment is inherently influenced by cognitive load, fatigue, and varying risk preferences. This contrasts sharply with the rigid, objective behavior assumed by classical performance metrics [4,5,6]. Given that human operators are increasingly utilized as primary decision-makers in diverse fields, such as medicine, surveillance, and crowdsourcing [3,7], and that the subjective experiences of human users are now a central consideration in the design of many modern systems [8,9,10,11,12], a fundamentally different analytical framework is required. Such a framework must accommodate the nuances of human participation, moving beyond the traditional models developed for detection systems comprised solely of physical sensors.

One of the most popular methods to incorporate the effects of a behavioral DA, i.e., a human, in detection systems is to adopt a performance metric that models human behavioral biases using prospect theory (PT), which is the most frequently used method since it captures decision-making behaviors of human DAs [13,14,15,16,17,18,19,20,21]. PT posits that behavioral biases arise from the inconsistent perception of objective probability and utility values. To model these perceptions, PT employs a probability weighting function and a utility valuation function [22,23,24,25,26]. By devising a performance metric that utilizes the perceived probability and utility values, it is possible to incorporate the effects of human DAs into the detection system. For example, a PT-based subjective utility function is used as a performance metric in [13,14,15,17,18]. The behavioral risk and behavioral utility have been employed as behavioral variants of the classical Bayes risk [16,19,20]. Additionally, a PT-based formulation of the NP problem was recently proposed in [21].

It has been established that, due to the non-linearity of the probability weighting function, direct analytical approaches often fail to reveal the structure of optimal detection rules for PT-based performance metrics. Moreover, it is reported that LRTs or likelihood ratio quantizers (LRQs), which are the canonical optimal rules for rational performance metrics, may be suboptimal for PT-based performance metrics [16,19,20,21]. As a remedy, a novel approach to characterize the optimal decision rules for any performance metric that can be expressed as a function of the *pairwise error probabilities* for *M*-ary hypothesis testing was developed in [16,19,20]. In this context, a pairwise error probability vector denotes the likelihood of selecting a specific hypothesis given that a different hypothesis is true. We represent these probabilities collectively as a single column vector, referred to throughout this study as the *pairwise probability vector*.

In this work, we provide a comprehensive review of the framework that was developed in our previous works with application to PT-based performance metrics. The presented framework is built on an indirect approach to characterize the optimal decision rules. Rather than performing optimization over the infinite-dimensional space of all possible randomized decision rules, the performance metric is optimized directly over the set of all achievable pairwise probability vectors. This shift is justified by the fact that decision rules influence performance metrics solely through their constituent pairwise error probabilities. By leveraging the compactness and convexity of this feasible set, the optimal decision rules can be characterized based on the geometric location of their corresponding pairwise probability vectors. Specifically, we demonstrate that any element within the set of all achievable pairwise probability vectors can be realized through a randomization of two LRQs constructed with different parameter sets. More explicitly, one of these LRQs can always be chosen as a deterministic LRQ, while the second may be either deterministic or randomized. In this context, a deterministic LRQ partitions the space of likelihood ratios into regions where a hypothesis is selected via convex separating hyperplanes. A randomized LRQ utilizes randomization only if the observation falls on a separating hyperplane where multiple hypotheses yield the same weighted cost. Furthermore, any randomized LRQ can be expressed as a randomization of deterministic LRQs that are constructed with the same parameter set but favor different hypotheses when an observation lies on a separating hyperplane. While this approach is particularly well-suited for non-linear PT-based performance metrics, the proposed method also provides a comprehensive toolkit for characterizing optimal decision rules for any metric that is a function of pairwise error probabilities.

In this paper, we also apply the proposed framework to a PT-based performance metric, namely the behavioral utility. The pairwise probability vectors of LRQs are fundamentally restricted to the boundary of the set of all achievable pairwise probability vectors. However, due to the non-linearity of the probability weighting function in PT, the behavioral utility may be maximized at a point residing in the interior of this feasible set. We demonstrate that by leveraging the randomized decision rules characterized in our framework, such interior points of the feasible set can be realized, whereas they remain inaccessible to standard deterministic LRQs. We corroborate these theoretical findings and demonstrate the efficacy of the proposed framework through several numerical examples, where the behavioral utility serves as the performance metric for the decision rules.

In this paper, we focus on the following points:

- We provide a general framework, which is developed in several papers [16,19,20], and present a unified approach for the characterization of optimal decision rules for any performance metric that can be expressed as a function of the pairwise probability vector. This framework leverages the geometric properties of the set of all achievable pairwise probability vectors. Specifically, we show that for *M*-ary hypothesis testing, any pairwise probability vector can be realized through a randomization of at most two (possibly randomized) LRQs constructed with different parameter sets.
- We present a method to sweep the set of all achievable pairwise probability vectors for the binary hypothesis-testing case.
- We apply the unified framework to a PT-based performance metric, namely the *behavioral utility*, for binary hypothesis-testing problems.

The remainder of this paper is organized as follows. In Section 2, the problem formulation for *M*-ary hypothesis testing is provided, and optimal decision rules for classical performance metrics, such as the Bayes risk and the NP criterion, are presented for review purposes. Section 3 combines the results of several works to form a framework for the characterization of optimal decision rules. Section 4 applies this framework to the PT-based performance metric, i.e., behavioral utility. The numerical results are provided in Section 5, and Section 6 concludes the paper.

## 2. Optimal Decision Rules for Classical Performance Metrics

In detection problems, the underlying state of a POI is determined by processing the observation of a DA by a decision rule. The observation space of the DA and the observation sampled from the observation space of the DA are denoted by Y and Y, respectively, where $Y\subseteq R^{N}$. The various possible states of the POI are represented by a finite set of *M* hypotheses, denoted as $H_{0},H_{1},\ldots ,H_{M-1}$. The probability distribution of the observation Y under each hypothesis is given by

$$\begin{array}{cc} H_{0} & :Y∼f_{0}\\ H_{1} & :Y∼f_{1}\\ \; & \vdots \\ H_{M-1} & :Y∼f_{M-1} \end{array}$$

where $f_{i}$ represents the probability density of Y under hypothesis $H_{i}$ with respect to some reference measure μ on Y. Depending on whether the observation space Y is continuous or discrete, $f_{j}$ denotes either a *probability density function (pdf)* or a *probability mass function (pmf)* under hypothesis $H_{j}$, respectively. For conciseness, the term *distribution* is used to refer to both pdfs and pmfs in the remainder of this paper.

The decision-making process of the DA is symbolized with

(1) q=δ(y)

where y is the DA’s observation (i.e., a realization of Y), δ is a possibly randomized decision rule employed by the DA and *q* is the DA’s decision. Given that y is observed, the decision rule δ is defined as the following vector: $\delta \left(y\right)={\left[\delta_{0}\left(y\right),\ldots ,\delta_{M-1}\left(y\right)\right]}^{⊤}$, where $\delta_{i}\left(y\right)$ denotes the probability of choosing $H_{i}$ given that y is observed for i∈{0,1,…,M−1}. Consequently, the probability of choosing hypothesis $H_{i}$ given that hypothesis $H_{j}$ is the true state can be computed as

(2) $$p_{ij}=\int_{Y}\delta_{i}\left(y\right)f_{j}\left(y\right)\;\mu \left(dy\right)\;,$$

where ∫μ(dy) denotes an integral operation if Y follows a continuous distribution and a summation over the observation space if Y follows a discrete distribution. These conditional probabilities in (2), often referred to as pairwise (error) probabilities, are utilized in the metrics that quantify the performance of the randomized decision rule δ∈Δ under each possible hypothesis, where Δ denotes the set of all possible (randomized) decision rules.

Note that deterministic decision rules can be viewed as a special case of randomized decision rules. Specifically, a deterministic decision rule partitions the observation space Y into *M* disjoint subsets ${\left\{Y_{i}\right\}}_{i=0}^{M-1}$. If the observation y falls into the subset $Y_{i}$, the hypothesis $H_{i}$ is selected deterministically [1]. This implies that for $y\in Y_{i}$, $\delta_{i}\left(y\right)=1$ and $\delta_{k}\left(y\right)=0$ for all k≠i. Consequently, for a deterministic decision rule, the pairwise probability of choosing $H_{i}$ when the true hypothesis is $H_{j}$ given in (2) simplifies to

(3) $$p_{ij}=\int_{Y_{i}}f_{j}\left(y\right)\;\mu \left(dy\right)\;.$$

The performance of a decision rule (detector) is evaluated using a performance metric function. In the literature, the most frequently utilized performance metrics include the Bayes risk, the NP criterion, the minimax criterion, and the restricted Bayes risk [1,2]. An optimal decision rule is defined as one that optimizes these respective performance metrics. In the following sections, we focus specifically on the Bayes risk and the NP criterion and present the derivations of the optimal decision rules.

### 2.1. Bayesian Hypothesis Testing

A detection problem in which the performance metric is the Bayes risk is referred to as the Bayesian hypothesis-testing problem. In this problem, the prior probabilities ${\left\{\pi_{j}\right\}}_{j=0}^{M-1}$ are assumed to be known, and the Bayes risk of a randomized decision rule δ∈Δ is expressed as

(4) $$r\left(\delta \right)=\sum_{j=0}^{M-1}\pi_{j}R_{j}\left(\delta \right)\;,$$

where $R_{j}\left(\delta \right)$ denotes the conditional risk under hypothesis $H_{j}$, which is defined as

(5) $$R_{j}\left(\delta \right)=\sum_{i=0}^{M-1}c_{ij}P\left(H_{i}|H_{j}\right)=\sum_{i=0}^{M-1}c_{ij}E_{j}\left[\delta_{i}\left(Y\right)\right]\;,$$

with $c_{ij}$ representing the finite cost incurred when hypothesis $H_{i}$ is selected given that $H_{j}$ is the true state. Here, $E_{j}\left[\cdot \right]$ denotes the expectation operator with respect to the distribution $f_{j}\left(y\right)$, and $E_{j}\left[\delta_{i}\left(Y\right)\right]$ corresponds to the pairwise probability of choosing $H_{i}$ given that $H_{j}$ is true, i.e., $p_{ij}=E_{j}\left[\delta_{i}\left(Y\right)\right]$. Thus, the Bayes risk represents the weighted average of the conditional risks, which themselves quantify the average cost incurred by employing decision rule δ when hypothesis $H_{j}$ is true.

The Bayes rule is the optimal decision rule that minimizes the Bayes risk. To obtain it, we first rewrite the Bayes risk by expanding the conditional probabilities:

(6) $$r\left(\delta \right)=\sum_{j=0}^{M-1}\pi_{j}\sum_{i=0}^{M-1}c_{ij}p_{ij}\;.$$

By substituting (2) into the expression above and interchanging the order of summation and integration, we obtain

(7) $$r\left(\delta \right)=\int_{Y}\sum_{i=0}^{M-1}\delta_{i}\left(y\right)\left(\sum_{j=0}^{M-1}\pi_{j}c_{ij}f_{j}\left(y\right)\right)\mu \left(dy\right)\;.$$

Let $f\left(y\right)≜\sum_{j=0}^{M-1}\pi_{j}f_{j}\left(y\right)$ denote the marginal μ(·)-density of the observation, and let $\pi_{j}\left(y\right)≜P\left(H_{j}|y\right)=\pi_{j}f_{j}\left(y\right)/f\left(y\right)$ represent the posterior probability that hypothesis $H_{j}$ is true given the observation y. The Bayes risk in (7) can then be expressed as

(8) $$r\left(\delta \right)=\int_{Y}\left(\sum_{i=0}^{M-1}\delta_{i}\left(y\right)\sum_{j=0}^{M-1}c_{ij}\pi_{j}\left(y\right)\right)f\left(y\right)\mu \left(dy\right)\;.$$

By defining the *posterior cost* of choosing hypothesis $H_{i}$ as

(9) $$c_{i}\left(y\right)≜\sum_{j=0}^{M-1}c_{ij}\pi_{j}\left(y\right),$$

the Bayes risk in (8) becomes

(10) $$\begin{array}{cc} r(\delta ) & =\int_{Y}\left(\sum_{i=0}^{M-1}\delta_{i}\left(y\right)c_{i}\left(y\right)\right)f\left(y\right)\mu \left(dy\right)\\ \; & \geq \int_{Y}\min_{0\leq i\leq M-1}\left\{c_{i}\left(y\right)\right\}f\left(y\right)\mu \left(dy\right)\;. \end{array}$$

The lower bound in (10) is achieved if, for every y∈Y, we set $\delta_{\ell }\left(y\right)\geq 0$ for $\ell \in C\left(y\right)≜\{j\in \left\{0,1,\ldots ,M-1\right\}:c_{j}\left(y\right)=\min_{i}c_{i}\left(y\right)\}$ such that $\sum_{\ell \in C(y)}\delta_{\ell }\left(y\right)=1$, and $\delta_{i}\left(y\right)=0$ for all i∉C(y). In other words, when multiple hypotheses attain the same minimum posterior cost for a given observation y, choosing a hypothesis from the set C(y) deterministically or selecting among them randomly yields an identical Bayes risk value. Consequently, randomized decision rules are unnecessary. Hence, the Bayes rule can be expressed by selecting an index ℓ∈C(y) and setting:

(11) $$\delta_{\ell }^{B}\left(y\right)=1,\;\mathrm{for}\;\mathrm{some}\;\ell \in C\left(y\right)\;,$$

with $\delta_{i}^{B}\left(y\right)=0$ for all i∈{0,1,…,M−1}\{ℓ}. Namely, any tie in posterior costs is broken deterministically in favor of a hypothesis $H_{\ell }$ such that ℓ∈C(y).

For the case of *uniform cost assignment (UCA)*, where the cost of a correct decision is $c_{ii}=0$ and the cost of any erroneous decision is $c_{ij}=1$ for i≠j, the Bayes decision rule reduces to the *maximum a posteriori (MAP)* decision rule as follows. Under UCA, the posterior cost in (9) simplifies to $c_{i}\left(y\right)=1-\pi_{i}\left(y\right)$; therefore, minimizing the posterior cost as in (11) becomes equivalent to maximizing the posterior probability. Hence, the MAP rule is given by

(12) $$\delta_{\ell }^{\mathrm{MAP}}\left(y\right)=1,\;\ell =\underset{0\leq i\leq M-1}{\arg\;\max}\;\pi_{i}\left(y\right)\;,$$

with $\delta_{k}^{\mathrm{MAP}}\left(y\right)=0$ for all k≠ℓ, where ties in argmax are broken in a deterministic fashion. Furthermore, if the prior probabilities are assumed to be equal (i.e., $\pi_{j}=1/M$ for all *j*), the MAP rule further reduces to the *maximum likelihood (ML)* decision rule:

(13) $$\delta_{\ell }^{\mathrm{ML}}\left(y\right)=1,\;\ell =\underset{0\leq i\leq M-1}{\arg\;\max}\;f_{i}\left(y\right)\;,$$

where $\delta_{k}^{\mathrm{ML}}\left(y\right)=0$ for all k≠ℓ.

#### Binary Hypothesis Testing

In binary hypothesis testing, the POI can be in one of M=2 states, denoted by $H_{0}$ and $H_{1}$, with prior probabilities $\pi_{0}$ and $\pi_{1}$, respectively. In this case, the optimal Bayes decision rule simplifies to a comparison of the posterior costs (cf. (11)):

(14) $$c_{0}\left(y\right)\overset{H_{0}}{\underset{H_{1}}{⋛}}c_{1}\left(y\right)\;,$$

which, by substituting the definitions of posterior cost in (9), can explicitly be written as

(15) $$c_{00}\pi_{0}\left(y\right)+c_{01}\pi_{1}\left(y\right)\overset{H_{0}}{\underset{H_{1}}{⋛}}c_{10}\pi_{0}\left(y\right)+c_{11}\pi_{1}\left(y\right)\;.$$

Assuming $c_{01}>c_{11}$ (i.e., the cost of an error exceeds the cost of a correct decision), we can substitute the posterior probabilities $\pi_{j}\left(y\right)=\pi_{j}f_{j}\left(y\right)/f\left(y\right)$, and by rearranging the terms, simplify the Bayes decision rule to a likelihood ratio test (LRT):

(16) $$L\left(y\right)≜\frac{f_{1}\left(y\right)}{f_{0}\left(y\right)}\overset{H_{1}}{\underset{H_{0}}{⋛}}\frac{\left(c_{10}-c_{00}\right)\pi_{0}}{\left(c_{01}-c_{11}\right)\pi_{1}}≜\eta \;,$$

where η denotes the optimal threshold against which the likelihood ratio is compared. The decision rule $\delta^{B}$ is then fully characterized by the indicator function $\delta_{1}^{B}\left(y\right)$:

(17) $$\delta_{1}^{B}\left(y\right)=\left\{\begin{array}{cc} 1, & \mathrm{if}\;L(y)\geq \eta \\ 0, & \mathrm{if}\;L(y)<\eta \;, \end{array}\right.$$

with $\delta_{0}^{B}\left(y\right)=1-\delta_{1}^{B}\left(y\right)$.

Similarly, for binary hypothesis testing, the MAP rule in (12) can be expressed in terms of the weighted likelihood functions:

(18) $$\pi_{1}f_{1}\left(y\right)\overset{H_{1}}{\underset{H_{0}}{⋛}}\pi_{0}f_{0}\left(y\right)\;,$$

which is equivalent to the following LRT:

(19) $$\delta_{1}^{\mathrm{MAP}}\left(y\right)=\left\{\begin{array}{cc} 1, & \mathrm{if}\;L\left(y\right)\geq \frac{\pi_{0}}{\pi_{1}}\\ 0, & \mathrm{otherwise}\;. \end{array}\right.$$

Assuming equal prior probabilities ($\pi_{0}=\pi_{1}=1/2$), the MAP decision rule further reduces to the ML decision rule, which compares the likelihood ratio to a unit threshold:

(20) $$\delta_{1}^{\mathrm{ML}}\left(y\right)=\left\{\begin{array}{cc} 1, & \mathrm{if}\;L(y)\geq 1\\ 0, & \mathrm{otherwise}\;. \end{array}\right.$$

### 2.2. Neyman–Pearson (NP) Hypothesis Testing

For binary hypothesis testing, the NP criterion is one of the most widely utilized performance metrics alongside the Bayes risk. In the NP framework, the prior probabilities are assumed to be unknown. The objective is to design a decision rule (test) that maximizes the detection probability while maintaining the false alarm probability below a pre-specified threshold α, where α∈[0,1] is referred to as the *level* of the test.

The optimal NP decision rule is obtained by solving the following constrained optimization problem:

(21) $$\begin{array}{cccc} \; & \underset{\delta \in \Delta }{\mathrm{maximize}} & \; & P_{D}\left(\delta \right)\\ \; & \mathrm{subject}\;\mathrm{to} & \; & P_{F}\left(\delta \right)\leq \alpha \end{array}$$

where $P_{D}\left(\delta \right)=P\left(H_{1}|H_{1}\right)=p_{11}$ and $P_{F}\left(\delta \right)=P\left(H_{1}|H_{0}\right)=p_{10}$ denote the *detection probability* and *false alarm probability* of the randomized decision rule δ, respectively. In statistics, $P_{D}$ and $P_{F}$ are often referred to as the *power* and *size* of the test. Thus, the NP approach seeks to find the *most powerful α-level test* for comparing $H_{0}$ against $H_{1}$ [1,2].

In the following, we present the NP lemma, which proves that the solution to the optimization problem in (21) is a randomized LRT of the form:

(22) $$\delta_{1}^{\mathrm{NP}}\left(y\right)=\left\{\begin{array}{cc} 1, & \mathrm{if}\;f_{1}\left(y\right)>\eta_{0}f_{0}\left(y\right)\\ \gamma_{0}, & \mathrm{if}\;f_{1}\left(y\right)=\eta_{0}f_{0}\left(y\right)\\ 0, & \mathrm{if}\;f_{1}\left(y\right)<\eta_{0}f_{0}\left(y\right) \end{array}\right.$$

where the threshold $\eta_{0}\geq 0$ and the randomization constant $\gamma_{0}\in \left[0,1\right]$ are chosen to satisfy the size constraint as $P_{F}\left(\delta^{\mathrm{NP}}\right)=\alpha$ ([1] Section II.D).

**Lemma** **1** (**Neyman–Pearson Lemma [1]**)**.** *For a binary hypothesis-testing problem with densities $f_{0}\left(y\right)$ and $f_{1}\left(y\right)$, and a given level α>0, the following statements are true:*

*Optimality: Let $\tilde{\delta }=\left({\tilde{\delta }}_{0},{\tilde{\delta }}_{1}\right)$ be any decision rule satisfying $P_{F}\left(\tilde{\delta }\right)\leq \alpha$, and let ${\tilde{\delta }}^{\prime }=\left({\tilde{\delta }}_{0}^{\prime },{\tilde{\delta }}_{1}^{\prime }\right)$ be of the form*
  (23) $$\begin{array}{c} {\tilde{\delta }}_{1}^{\prime }\left(y\right)=\left\{\begin{array}{cc} 1, & \mathrm{if}\;\;\;\;f_{1}\left(y\right)>\eta \;f_{0}\left(y\right)\\ \gamma (y), & \mathrm{if}\;\;\;\;f_{1}\left(y\right)=\eta \;f_{0}\left(y\right)\\ 0, & \mathrm{if}\;\;\;\;f_{1}\left(y\right)<\eta \;f_{0}\left(y\right) \end{array}\right. \end{array}$$
  *where η≥0 and γ(y)∈[0,1] are such that $P_{F}\left({\tilde{\delta }}^{\prime }\right)=\alpha$. Then, $P_{D}\left({\tilde{\delta }}^{\prime }\right)\geq P_{D}\left(\tilde{\delta }\right)$. Hence, size-α decision rule of the form* (23) *is an NP rule.*
*Existence: For every α∈(0,1), there is a decision rule ${\tilde{\delta }}^{N}=\left({\tilde{\delta }}_{0}^{N},{\tilde{\delta }}_{1}^{N}\right)$ of the form of* (23) *with $\gamma \left(y\right)=\gamma_{0}$ (a constant) for which $P_{F}\left({\tilde{\delta }}^{N}\right)=\alpha$.*
*Uniqueness: Suppose ${\tilde{\delta }}^{\prime \prime }=\left({\tilde{\delta }}_{0}^{\prime \prime },{\tilde{\delta }}_{1}^{\prime \prime }\right)$ is any α-level NP rule for $H_{0}$ versus $H_{1}$. Then, ${\tilde{\delta }}_{1}^{\prime \prime }$ must be of the form of* (23) *except possibly on a subset of Y having zero probability under $H_{0}$ and $H_{1}$.*

The NP rule in (22) can be interpreted as a convex combination of two deterministic LRTs, $\delta^{*}=\left(\delta_{0}^{*},\delta_{1}^{*}\right)$ and $\delta^{†}=\left(\delta_{0}^{†},\delta_{1}^{†}\right)$, defined as follows:

(24) $$\delta_{1}^{*}\left(y\right)=\left\{\begin{array}{cc} 1, & \mathrm{if}\;L\left(y\right)\geq \eta_{0}\\ 0, & \mathrm{if}\;L\left(y\right)<\eta_{0}\;, \end{array}\right.\;\delta_{1}^{†}\left(y\right)=\left\{\begin{array}{cc} 1, & \mathrm{if}\;L\left(y\right)>\eta_{0}\\ 0, & \mathrm{if}\;L\left(y\right)\leq \eta_{0}\;. \end{array}\right.$$

Specifically, the randomized NP rule in (22) corresponds to selecting the deterministic rule $\delta^{*}$ with probability $\gamma_{0}$ and the rule $\delta^{†}$ with probability $1-\gamma_{0}$. Note that if the event $\left\{f_{1}\left(y\right)=\eta_{0}f_{0}\left(y\right)\right\}$ occurs with probability zero under both hypotheses (as is typical for continuous distributions), the randomization constant $\gamma_{0}$ becomes irrelevant, and the rule simplifies to a standard deterministic LRT.

## 3. Optimal Decision Rules Under General Criterion in Terms of Error Probabilities

In this section, we present a method to characterize optimal decision rules for performance metrics that are functions of pairwise error probabilities. A *generic decision criterion* expressed in terms of pairwise error probabilities can be formulated as the following optimization problem [19]:

(25) $$\begin{array}{cccc} \; & \underset{\delta \in \Delta }{\mathrm{minimize}} & \; & g_{0}\left(p\left(\delta \right)\right)\\ \; & \mathrm{subject}\;\mathrm{to} & \; & g_{i}\left(p\left(\delta \right)\right)\leq 0,\;i=1,2,\ldots ,m\\ \; & \; & & h_{j}\left(p\left(\delta \right)\right)=0,\;j=1,2,\ldots ,n \end{array}$$

where Δ is the set of all possible (randomized) decision rules, and $g_{i}$ and $h_{j}$ represent arbitrary functions of the pairwise error probabilities. The vector p(δ) denotes the pairwise probability vector, defined as

(26) $$p\left(\delta \right)={\left[p_{10},p_{20},\ldots ,p_{M-1\;0},p_{01},p_{21},\ldots ,p_{M-2\;M-1}\right]}^{⊤}\;.$$

where $p_{ij}$ denotes the probability of choosing $H_{i}$ when the true hypothesis is $H_{j}$, as defined in (2). Note that it is sufficient to include only the pairwise error probabilities (i.e., $p_{ij}$ for i≠j, i,j∈{0,1,…,M−1}) in p(δ), as the correct decision probabilities are linearly dependent: $p_{jj}=1-\sum_{i\neq j}p_{ij}$. Classical hypothesis-testing criteria, including Bayesian, minimax, NP, and restricted Bayesian, can all be viewed as special cases of the formulation in (25).

Decision rules influence performance metrics exclusively through their corresponding pairwise probability vectors. Consequently, optimizing a performance metric over the set of all achievable pairwise probability vectors is equivalent to optimizing it over the set of all randomized decision rules. We define the set of all achievable pairwise probability vectors as

(27) P(Δ)≜{p(δ):δ∈Δ}.

It is important to note that the set P(Δ) is both convex and compact, as established in [19,20,27]. Using this property, the general decision criterion in (25) can be reformulated as an optimization over the achievable set of pairwise probability vectors:

(28) $$\begin{array}{cccc} \; & \underset{p\in P(\Delta )}{\mathrm{minimize}} & \; & g_{0}\left(p\right)\\ \; & \mathrm{subject}\;\mathrm{to} & \; & g_{i}\left(p\right)\leq 0,\;i=1,2,\ldots ,m\\ \; & \; & & h_{j}\left(p\right)=0,\;j=1,2,\ldots ,n \end{array}$$

Next, we leverage the convexity and compactness of P(Δ) to elucidate the relationship between pairwise probability vectors and their corresponding decision rules. To develop the framework, we first consider a linear objective of the form $v^{⊤}p$, that is, a weighted sum of the pairwise error probabilities, and show that the corresponding optimal rules are likelihood ratio quantizers (LRQs) associated with vector v. We then return to the general optimization problem in (28) and use this characterization to describe the structure of the optimal decision rules.

Consider the unconstrained performance metric $v^{⊤}p\left(\delta \right)$, where $v\in R^{M(M-1)}$ is a weight vector with real-valued entries $v_{ij}$ for i,j∈{0,1,…,M−1} and i≠j. Since P(Δ) is a convex and compact set, for any v, there exists a supporting hyperplane $\left\{p:v^{⊤}p=v^{⊤}p^{*}\right\}$ such that $p^{*}\in P\left(\Delta \right)$ and $v^{⊤}p\geq v^{⊤}p^{*}$ for all p∈P(Δ) [28]. To derive the decision rule that yields $v^{⊤}p^{*}$, we express the performance metric $v^{⊤}p\left(\delta \right)$ as follows:

(29) $$v^{⊤}p\left(\delta \right)=\sum_{i=0}^{M-1}\sum_{j=0,j\neq i}^{M-1}v_{ij}p_{ij}\;.$$

By substituting $p_{ij}$ with (2) and interchanging the order of summation and integration, (29) becomes

(30) $$v^{⊤}p\left(\delta \right)=\int_{Y}\sum_{i=0}^{M-1}\delta_{i}\left(y\right)\left(\sum_{j=0,j\neq i}^{M-1}v_{ij}f_{j}\left(y\right)\right)\mu \left(dy\right)\;.$$

By defining the *weighted likelihood sum* for hypothesis $H_{i}$ as

(31) $$V_{i}\left(y\right)≜\sum_{j=0,j\neq i}^{M-1}v_{ij}f_{j}\left(y\right),$$

the performance metric in (30) becomes

(32) $$\begin{array}{cc} v^{⊤}p\left(\delta \right) & =\int_{Y}\sum_{i=0}^{M-1}\delta_{i}\left(y\right)V_{i}\left(y\right)\;\mu \left(dy\right)\\ \; & \geq \int_{Y}\min_{0\leq i\leq M-1}V_{i}\left(y\right)\;\mu \left(dy\right)\;. \end{array}$$

The lower bound in (32); hence, the minimizer of the performance metric, can be achieved by a randomized LRQ or deterministic LRQ as specified below.

Let V(y) denote the set of indices that minimizes $V_{i}\left(y\right)$ in (32). That is,

$$V\left(y\right)≜\left\{j\in \left\{0,1,\ldots ,M-1\right\}:V_{j}\left(y\right)=\min_{0\leq i\leq M-1}V_{i}\left(y\right)\right\}.$$

We call a decision rule δ a *randomized LRQ associated with v* if

(33) $$\sum_{\ell \in V(y)}\delta_{\ell }\left(y\right)=1$$

and $\delta_{i}\left(y\right)=0$ for all i∉V(y). It is noted that any randomized LRQ associated with v attains the lower bound in (32), and hence any such rule is optimal for minimizing $v^{⊤}p\left(\delta \right)$. As a special case of randomized LRQ, we can set one of the $\delta_{\ell }\left(y\right)$ components to one for a certain *ℓ* in V(y) and set all the other elements of δ to zero, yielding a deterministic LRQ. In other words, we call a decision rule δ a *deterministic LRQ associated with v* if

(34) $$\delta_{\ell }\left(y\right)=1\;\mathrm{for}\;\mathrm{some}\;\ell \in V\left(y\right)$$

and $\delta_{i}\left(y\right)=0$ for all i∈{0,1,…,M−1}∖{ℓ}. Therefore, every deterministic LRQ associated with v is also optimal for the same linear objective, $v^{⊤}p\left(\delta \right)$.

**Remark** **1.** *In this context, a deterministic LRQ is a decision rule that separates the likelihood ratio space into M convex polytopes in which one hypothesis is chosen deterministically. The polytopes in which different hypotheses are selected are separated via convex hyperplanes. It should be noted that, if the observation y corresponds to a point on a separating hyperplane on the likelihood ratio space, there exists more than one element in the V(y) set and a deterministic LRQ selects a hypothesis deterministically as given in* (34)*. Conversely, a randomized LRQ chooses a hypothesis randomly from the hypotheses whose indices compose the V(y) set, if the likelihood ratio of observation, y, corresponds to a separating hyperplane. Naturally, a randomized LRQ can be viewed as a randomization of the deterministic LRQs constructed with the same parameter set, v.*

Next, we consider the general optimization problem in (28). We define the set of *boundary points* as a subset of the observation space Y for a given weight vector v as [19]

(35) $$B\left(v\right)≜\underset{\begin{array}{c} 0\leq i<j\leq M-1 \end{array}}{⋃}B_{i,j}\left(v\right)\;,$$

where each subset $B_{i,j}\left(v\right)$ represents the points where at least two hypotheses achieve the same minimum weighted likelihood sum:

(36) $$B_{i,j}\left(v\right)≜\left\{y\in Y:V_{i}\left(y\right)=V_{j}\left(y\right)\leq V_{k}\left(y\right),\forall k\neq i,j\right\}\;.$$

The complement of the boundary set, denoted as $\bar{B}\left(v\right)$, contains the observations where a single hypothesis strictly minimizes the weighted likelihood sum; that is,

(37) $$\begin{array}{cc} \bar{B}\left(v\right)=Y\setminus B\left(v\right)=\{y\in Y\;:\;V_{i}\left(y\right) & <V_{j}\left(y\right),\;\mathrm{for}\;\mathrm{some}\;\\ & 0\leq i\leq M-1\;\mathrm{and}\;\mathrm{all}\;0\leq j\leq M-1,\;j\neq i\}\;. \end{array}$$

If the boundary set B(v) has zero probability measure under all hypotheses, as is typical for continuous distributions, ties can be broken arbitrarily without affecting the performance metric. However, if B(v) occurs with non-zero probability (e.g., in discrete or mixed distributions), the specific hypothesis selected when multiple indices yield identical $V_{i}\left(y\right)$ values directly influences the resulting pairwise error probabilities.

In ([19] Lemma), the decision rules which yield pairwise probability vectors that reside on the boundary of P(Δ) are characterized. We summarize this lemma below:

**Lemma** **2** ([19] Lemma)**.** *Let $p^{*}$ be a point on the boundary of P(Δ), and let $\left\{p:v^{⊤}p=v^{⊤}p^{*}\right\}$ be a supporting hyperplane to P(Δ) at $p^{*}$.*

- ***Case 1 (Zero Probability Boundary):*** *If the boundary set B(v) defined in* (35) *has zero probability measure under all hypotheses, then any deterministic LRQ associated with v of the form in* (34) *yields the pairwise probability vector $p^{*}$.*
- ***Case 2 (Non-zero Probability Boundary):*** *If B(v) has non-zero probability measure under at least one hypothesis, then $p^{*}$ is achievable by a randomized LRQ associated with v as defined in* (33)*, which randomizes at most M(M−1) deterministic LRQs, all of which take the form of* (34) *and correspond to the same weight vector v.*

In Case 1 of Lemma 2, the optimal decision rule for $\inf_{p\in P(\Delta )}v^{⊤}p$ is unique up to sets of probability measure zero. Consequently, the corresponding optimal pairwise probability vector $p^{*}$ is the unique minimizer of the linear objective. It follows that $p^{*}$ is an exposed point of P(Δ), and hence also an extreme point. Since $p^{*}$ is an extreme point of P(Δ), it cannot be written as a nontrivial convex combination of two distinct points in P(Δ). Therefore, a deterministic LRQ in (34) yields $p^{*}$.

In Case 2 of Lemma 2, the boundary of P(Δ) may contain points that are not extreme points, meaning they can be expressed as a convex combination of the extreme points in the set. Consequently, the linear functional $v^{⊤}p$ may not have a unique minimizer. All pairwise probability vectors that minimize $v^{⊤}p\left(\delta \right)$ lie on the intersection of P(Δ) with the supporting hyperplane $\left\{p:v^{⊤}p=v^{⊤}p_{0}\right\}$, which has a dimension of at most M(M−1)−1. By *Carathéodory’s Theorem*, any point $p^{*}$ within this intersection can be represented as a convex combination of at most M(M−1) extreme points of the set [29]. These extreme points correspond to deterministic decision rules of the form (34) associated with the same weight vector v, [27]. Crucially, these deterministic rules differ only in the specific hypothesis selected for observations y∈B(v), where the weighted likelihood sums are tied.

**Figure 1:**
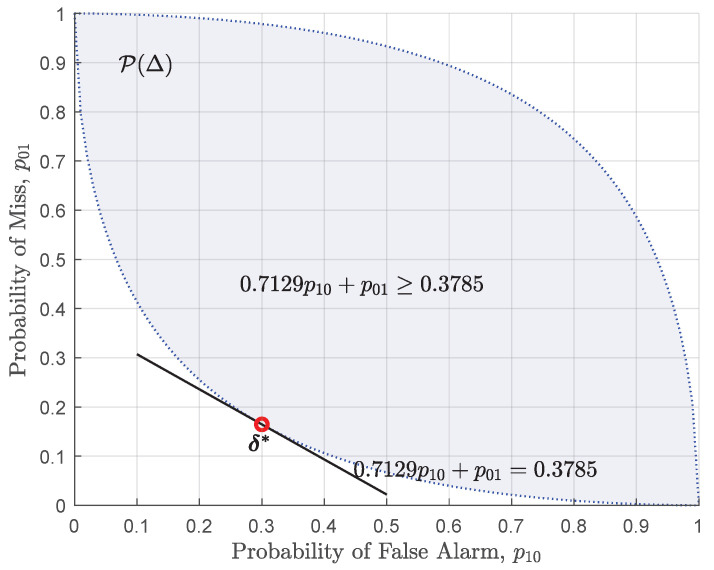
Set of achievable pairwise probability vectors when Y has a continuous distribution (Lemma 2-Case 1).

**Figure 2:**
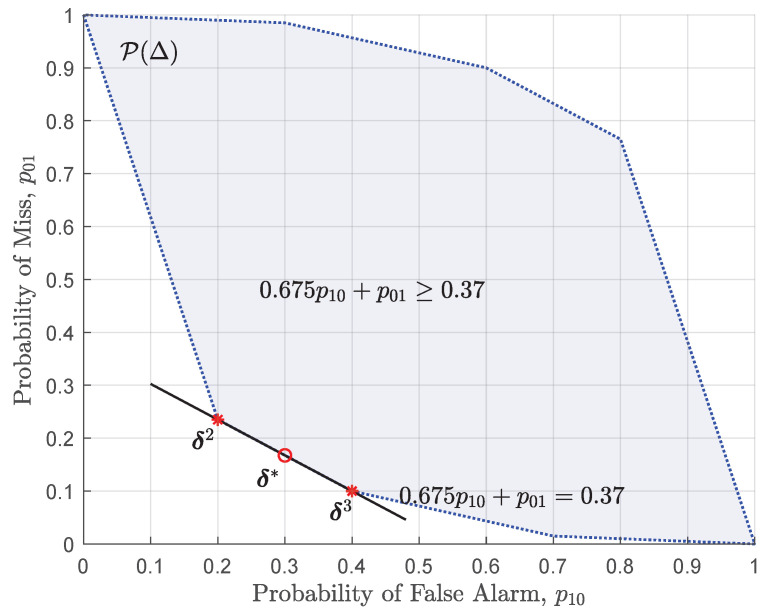
Set of achievable pairwise probability vectors when Y has a discrete distribution (Lemma 2-Case 2).

In Figure 1 and Figure 2, we provide illustrative examples for both cases of Lemma 2 within a binary hypothesis-testing framework. The set of all achievable pairwise probability vectors, P(Δ), is highlighted in the figures.

First, we consider the case in which the boundary set B(v) has zero probability measure. Let the weights be assigned as $v_{01}=1$ and $v_{10}=0.7129$. Under these weights, the weighted likelihood sums in (31) become $V_{0}\left(y\right)=v_{01}f_{1}\left(y\right)=f_{1}\left(y\right)$ and $V_{1}\left(y\right)=v_{10}f_{0}\left(y\right)=0.7129f_{0}\left(y\right)$. Since B(v) has zero probability measure, the probability of $V_{0}\left(y\right)=V_{1}\left(y\right)$ occur with zero probability. Hence, $v^{⊤}p$ is minimized if the optimal decision rule selects the hypothesis that yields the minimum $V_{i}\left(y\right)$ value, which can be written as

(38) $$\delta_{\ell }^{*}\left(y\right)=1,\;\mathrm{for}\;\ell =\underset{i\in \{0,1\}}{\arg\;\min}V_{i}\left(y\right)\;.$$

More explicitly, for binary hypothesis testing, the decision rule given above can be written as

(39) $$\begin{array}{cc} v_{01}f_{1}\left(y\right) & \overset{H_{0}}{\underset{H_{1}}{⋛}}v_{10}f_{0}\left(y\right)\;. \end{array}$$

Since $v_{01}$ and $v_{10}$ are positive, the decision rule $\delta^{*}$ can be expressed as

(40) $$L\left(y\right)=\frac{f_{1}\left(y\right)}{f_{0}\left(y\right)}⋛\frac{v_{10}}{v_{01}}\;.$$

More explicitly, the optimal decision rule that minimizes $v^{⊤}p\left(\delta \right)$ is

(41) $$\delta_{1}^{*}\left(y\right)=\left\{\begin{array}{cc} 1, & \mathrm{if}\;L(y)\geq 0.7129,\\ 0, & \mathrm{otherwise}\;. \end{array}\right.$$

Since the boundary points of y occur with zero probability measure, the hypothesis selected when L(y)=0.7129 does not change the pairwise probabilities. The resulting pairwise probability vector for this rule is $p^{*}={\left[0.3,0.1646\right]}^{⊤}$, which achieves the minimum performance score of $v^{⊤}p^{*}\left(\delta^{*}\right)=0.3785$. The optimal pairwise probability vector, $p^{*}\left(\delta^{*}\right)$ is at the intersection of P(Δ) and the hyperplane $0.7129p_{10}+p_{01}=0.3785$, as shown in Figure 1.

The boundary of the set of pairwise probability vectors for an example to Case 2 of Lemma 2 is shown by the dotted lines in Figure 2. In that case, the weights are set as $v_{10}=0.675$ and $v_{01}=1$, which lead to the weighted likelihood sums $V_{0}\left(y\right)=f_{1}\left(y\right)$ and $V_{1}\left(y\right)=0.675f_{0}\left(y\right)$. The optimal decision rule selects the hypothesis with minimum $V_{i}\left(y\right)$ value for i∈{0,1}, which can be written as

(42) $$\begin{array}{cc} v_{01}f_{1}\left(y\right) & \overset{H_{0}}{\underset{H_{1}}{⋛}}v_{10}f_{0}\left(y\right)\;. \end{array}$$

Since B(v) occur with nonzero probability, the probability of $v_{01}f_{1}\left(y\right)$ and $v_{10}f_{0}\left(y\right)$ being equal has a nonzero probability for these specific weights. Hence, there exist infinitely many pairwise probability vectors that minimize $v^{⊤}p\left(\delta \right)$, which compose the set that is defined as the intersection of the P(Δ) set and the hyperplane (line) given below:

(43) $$0.675p_{10}+p_{01}=0.37\;.$$

These decision rules differ only in the probability with which $H_{1}$ is selected when $v_{01}f_{1}\left(y\right)=v_{10}f_{0}\left(y\right)$, a condition that can be equivalently expressed as $L\left(y\right)=\frac{v_{10}}{v_{01}}$. The deterministic decision rules $\delta^{2}$ and $\delta^{3}$, whose pairwise probability vectors are the extreme points of the set of minimizers for $v^{⊤}p\left(\delta \right)$, are given by

(44) $$\delta_{1}^{2}\left(y\right)=\left\{\begin{array}{cc} 1, & \mathrm{if}\;L(y)>0.675,\\ 0, & \mathrm{otherwise}\;, \end{array}\right.\;\mathrm{and}\;\delta_{1}^{3}\left(y\right)=\left\{\begin{array}{cc} 1, & \mathrm{if}\;L(y)\geq 0.675,\\ 0, & \mathrm{otherwise}\;. \end{array}\right.$$

The pairwise probability vectors of $\delta^{2}$ and $\delta^{3}$ are shown in Figure 2. Any point $p^{*}\left(\delta^{*}\right)$ on this segment can be expressed as the convex combination:

(45) $$p^{*}\left(\delta^{*}\right)=\lambda p^{2}\left(\delta^{2}\right)+\left(1-\lambda \right)p^{3}\left(\delta^{3}\right)\;,$$

where λ∈[0,1]. The randomized decision rule $\delta^{*}$ that achieves $p^{*}\left(\delta^{*}\right)$ is similarly a convex combination $\delta^{*}=\lambda \delta^{2}+\left(1-\lambda \right)\delta^{3}$, which can be explicitly written as a randomized LRT:

(46) $$\delta_{1}^{*}\left(y\right)=\left\{\begin{array}{cc} 1, & \mathrm{if}\;L(y)>0.675,\\ 1-\lambda , & \mathrm{if}\;L(y)=0.675,\\ 0, & \mathrm{if}\;L(y)<0.675\;. \end{array}\right.$$

In this case, if the event {L(y)=0.675} occurs with non-zero probability, hypothesis $H_{1}$ is selected with probability 1−λ to satisfy the specific performance requirement.

Based on the characterization of the decision rules whose pairwise probability vectors lie on the boundary of P(Δ), the following theorem, developed in ([19] Theorem), provides a complete characterization of the optimal decision rules according to the location of the optimizer in P(Δ), including interior points that are not covered directly by Lemma 2.

**Theorem** **1** ([19] Theorem)**.** *Suppose the optimization problem in* (25) *is feasible, and let $\delta^{*}$ and $p^{*}\left(\delta^{*}\right)$ denote an optimal decision rule and its corresponding pairwise probability vector, respectively. An optimal decision rule that solves* (25) *can be characterized as follows:*

- *Case 1: If the boundary set B(v) defined in* (35) *has zero probability measure under all hypotheses for any weight vector v, then $\delta^{*}$ is a randomization among at most two deterministic LRQs of the form given in* (34)*, each specified by a distinct weight vector.*
- *Case 2: Otherwise, $\delta^{*}$ is a randomization of two LRQs with separate v vectors. Note that one of these LRQs can always be chosen as a deterministic LRQ of the form given in* (34)*, which corresponds to Case 1 of Lemma 2. The other LRQ is possibly a randomized LRQ that corresponds to Case 2 of Lemma 2.*

When the optimization problem in (25) exhibits specific structural properties, the maximum number of deterministic decision rules required to achieve optimal performance may be reduced below the bounds established in this theorem.

If the optimal pairwise probability vector $p^{*}$ resides on the boundary of P(Δ), Lemma 2 directly characterizes the optimal decision rule. Specifically, if the boundary set B(v) has a zero (Case 1) or non-zero (Case 2) probability measure, $p^{*}\left(\delta^{*}\right)$ is achieved by a deterministic LRQ or (possibly) a randomized LRQ, respectively. In the latter case, the LRQ can be written as a randomization among at most M(M−1) rules that are constructed using the same weight vector v [19].

**Figure 3:**
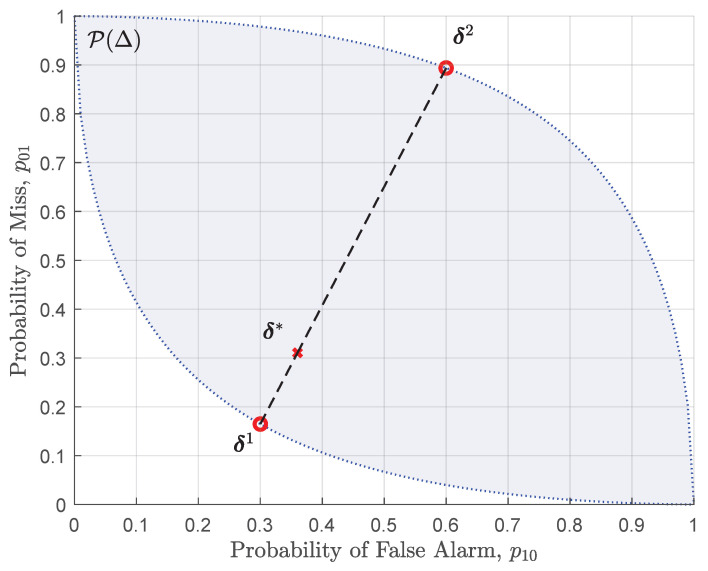
Set of achievable pairwise probability vectors and an example of Theorem 1 for Y with a continuous distribution (Theorem 1-Case 1).

Conversely, if the optimal pairwise probability vector resides in the interior of P(Δ), a randomization of two LRQs is required. Firstly, let B(v) have a zero probability measure. Under this assumption, the boundary of P(Δ) consists entirely of extreme points, which are achieved by the deterministic rules defined in (34). The optimal decision rule that attains $p^{*}\left(\delta^{*}\right)$ is a randomization of two deterministic LRQs characterized by different weight vectors. To see this, let $p^{1}\left(\delta^{1}\right)$ be a point on the boundary achieved by rule $\delta^{1}$ with weight vector $v^{1}$. A ray originating from $p^{1}\left(\delta^{1}\right)$ and passing through $p^{*}\left(\delta^{*}\right)$ intersects the boundary at another point $p^{2}\left(\delta^{2}\right)$. Since $p^{2}\left(\delta^{2}\right)$ is also an extreme point, it corresponds to a deterministic rule $\delta^{2}$ with weight vector $v^{2}$. Thus, $p^{*}\left(\delta^{*}\right)$ can be expressed as the convex combination:

(47) $$p^{*}\left(\delta^{*}\right)=\lambda p^{1}\left(\delta^{1}\right)+\left(1-\lambda \right)p^{2}\left(\delta^{2}\right)\;,$$

where $\lambda \in \left[0,1\right]$ is the randomization coefficient. The corresponding optimal decision rule is then

(48) $$\delta^{*}=\lambda \delta^{1}+\left(1-\lambda \right)\delta^{2}\;,$$

where $\delta^{1}$ and $\delta^{2}$ are selected with probabilities λ and 1−λ, respectively. An example for binary hypothesis testing is illustrated in Figure 3.

Now, assume that B(v) has a non-zero probability measure (Theorem 1, Case 2) and the $p^{*}$ is in the interior of P(Δ). The optimal pairwise probability vector $p^{*}\left(\delta^{*}\right)$ is achieved via a randomization between two LRQs with different weight vectors; one of these LRQs can always be chosen as a deterministic LRQ. To show this, let $p^{1}\left(\delta^{1}\right)$ be an extreme point of P(Δ) achieved by decision rule $\delta^{1}$ characterized by the weight vector $v^{1}$. A ray originating from $p^{1}\left(\delta^{1}\right)$ and passing through $p^{*}\left(\delta^{*}\right)$ intersects the boundary of P(Δ) at a point $\tilde{p}$. Since points on the boundary are not necessarily extreme points in Case 2, $\tilde{p}$ can be expressed as a convex combination of at most M(M−1) extreme points lying on the same supporting hyperplane. Let these points be $p^{2}\left(\delta^{2}\right),\ldots ,p^{M(M-1)+1}\left(\delta^{M(M-1)+1}\right)$, achieved by deterministic LRQs $\delta^{2},\ldots ,\delta^{M(M-1)+1}$. The optimal pairwise probability vector $p^{*}\left(\delta^{*}\right)$ can then be written as a convex combination of these M(M−1)+1 extreme points:

(49) $$p^{*}\left(\delta^{*}\right)=\sum_{k=1}^{M(M-1)+1}\lambda_{k}p^{k}\left(\delta^{k}\right)\;,$$

where $\lambda_{k}\in \left[0,1\right]$ and $\sum_{k=1}^{M(M-1)+1}\lambda_{k}=1$. Consequently, the optimal decision rule is a randomized LRQ, which can be written as a randomization of at most M(M−1)+1 deterministic rules as given below:

(50) $$\delta^{*}=\sum_{k=1}^{M(M-1)+1}\lambda_{k}\delta^{k}\;,$$

where each deterministic LRQ, $\delta^{k}$, is employed with probability $\lambda_{k}$. Notably, since the pairwise probability vectors $p^{2}\left(\delta^{2}\right),\ldots ,p^{M(M-1)+1}\left(\delta^{M(M-1)+1}\right)$ lie on the same supporting hyperplane, their corresponding decision rules can be constructed using the same weight vector v.

**Figure 4:**
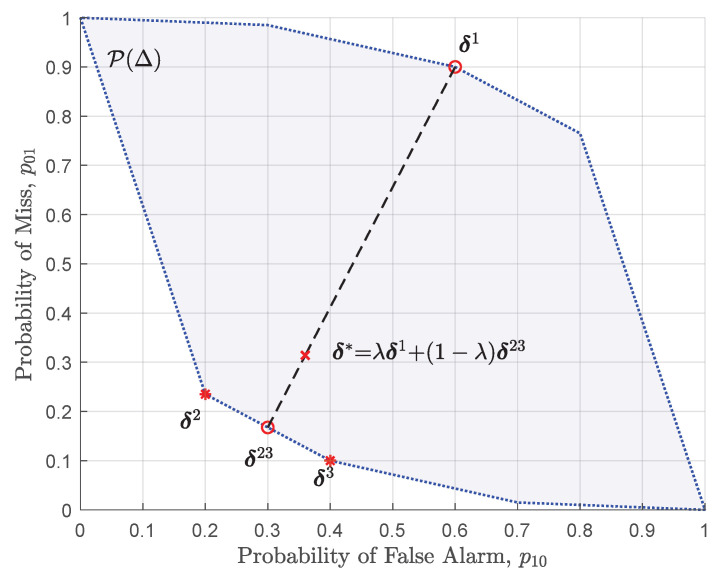
Set of achievable pairwise probability vectors and an example of Theorem 1 for Y with a discrete distribution (Theorem 1-Case 2).

An example illustrating Case 2 of Theorem 1 in a binary hypothesis-testing scenario is depicted in Figure 4 for binary hypothesis testing. Note that LRQs become LRTs for binary hypothesis testing. In this instance, the optimal decision rule is a randomization of two LRQs, $\delta^{1}$ and $\delta^{23}$. The pairwise probability vector of $\delta^{1}$ is an extreme point; hence, $\delta^{1}$ is a deterministic LRQ. On the other hand, the pairwise probability vector of $\delta^{23}$ reside at the line segment between the two extreme points that correspond to the deterministic LRQs, $\delta^{2}$ and $\delta^{3}$. Hence, the pairwise probability vector of $\delta^{23}$ can be written as a convex combination of these extreme points and thus, $\delta^{23}$ is a randomized LRQ that randomizes $\delta^{2}$ and $\delta^{3}$. Note that, since the pairwise probability vectors corresponding to $\delta^{2}$ and $\delta^{3}$ lie on the same supporting hyperplane, these two rules are constructed using the same weight vector v. The only distinction between $\delta^{2}$ and $\delta^{3}$ is the specific hypothesis selected when the likelihood ratio exactly equals the threshold value. This event occurs with non-zero probability in this case. It should be noted that the decision rule, $\delta^{1}$, can always be selected such that it corresponds to an extreme point on a different part of the boundary and is therefore characterized by a different weight vector.

### Special Cases

In this section, we demonstrate that optimal decision rules for standard performance metrics, such as Bayes risk and the NP criterion, can be characterized as special cases of the framework presented in the preceding part.

Bayesian *M*-ary Hypothesis Testing:

We first consider the *Bayesian M-ary hypothesis-testing* problem. By rearranging the summation terms, the Bayes risk in (6) can be expressed as a linear function of the pairwise probability vector:

(51) $$\begin{array}{cc} r(\delta ) & =\sum_{j=0}^{M-1}\sum_{\begin{array}{c} i=0\\ i\neq j \end{array}}^{M-1}\pi_{j}\left(c_{ij}-c_{jj}\right)p_{ij}+\sum_{j=0}^{M-1}\pi_{j}c_{jj}\\ \; & =v^{⊤}p\left(\delta \right)+\mathrm{constant}\;, \end{array}$$

where the weights are defined as $v_{ij}≜\pi_{j}\left(c_{ij}-c_{jj}\right)$. As established in the previous section, the decision rule that minimizes a performance metric of the form $v^{⊤}p\left(\delta \right)$ can be expressed as the deterministic rule defined in (34).

Binary Bayesian Hypothesis-Testing

For binary hypothesis testing (M=2), the decision rule in (34) simplifies to the following comparison:

(52) $$V_{1}\left(y\right)\overset{H_{1}}{\underset{H_{0}}{≷}}V_{0}\left(y\right)\;.$$

Substituting $V_{0}\left(y\right)=v_{01}f_{1}\left(y\right)$ and $V_{1}\left(y\right)=v_{10}f_{0}\left(y\right)$, the optimal Bayesian rule becomes a standard LRT:

(53) $$\delta_{1}^{B}\left(y\right)=\left\{\begin{array}{cc} 1, & \mathrm{if}\;L\left(y\right)\geq \frac{v_{10}}{v_{01}},\\ 0, & \mathrm{otherwise}\;, \end{array}\right.$$

where $v_{10}=\pi_{0}\left(c_{10}-c_{00}\right)$ and $v_{01}=\pi_{1}\left(c_{01}-c_{11}\right)$. Next, we focus on the probability of error performance metric, which can be viewed as a special case of Bayes risk with $c_{10}=c_{01}=1$ and $c_{00}=c_{11}=0$. We derive the lower bound for the probability of error and show that the lower bound can be achieved via the MAP rule.

**Remark** **2.** *Consider the scenario in which the performance metric is the minimum probability of error. The probability of error associated with the decision rule **δ** can be expressed as*

(54) $$P_{e}\left(p\left(\delta \right)\right)=\pi_{1}p_{01}+\pi_{0}p_{10}\;.$$

*The lower bound on the probability of error is given by*

(55) $$P_{e}\left(p\left(\delta \right)\right)\geq \pi_{1}-d_{v}\left(\pi_{0}f_{0}\left(y\right),\pi_{1}f_{1}\left(y\right)\right)\;,$$

*where $d_{v}\left(\pi_{0}f_{0}\left(y\right),\pi_{1}f_{1}\left(y\right)\right)$ denotes the total variation distance and is computed as [30]:*

(56) $$d_{v}\left(\pi_{0}f_{0}\left(y\right),\pi_{1}f_{1}\left(y\right)\right)=\underset{\delta \in \Delta }{\sup}\;\left[\pi_{1}p_{11}-\pi_{0}p_{10}\right]\;.$$

*The equality in* (55) *is achieved if the decision rule that yields the minimum probability of error is employed. This optimal decision rule can be obtained by solving the following optimization problem:*

(57) $$\delta^{*}=\underset{\delta \in \Delta }{\arg\;\min}\;P_{e}\left(p\left(\delta \right)\right)\;.$$

*Since the probability of error is a function of the pairwise probabilities, this optimization problem can equivalently be expressed as*

(58) $$p^{*}\left(\delta^{*}\right)=\underset{p\in P(\Delta )}{\arg\;\min}\;P_{e}\left(p\right)\;.$$

*By denoting $v={\left[\pi_{0},\pi_{1}\right]}^{T}$, the probability of error can compactly be written as $P_{e}\left(p\left(\delta \right)\right)=v^{T}p\left(\delta \right)$. Consequently, the decision rule that minimizes the probability of error and achieves the lower bound in* (55) *is the LRT given by*

(59) $$\delta^{*}\left(y\right)=\left\{\begin{array}{cc} 1, & \mathrm{if}\;L\left(y\right)\geq \frac{\pi_{0}}{\pi_{1}},\\ 0, & \mathrm{otherwise}\;. \end{array}\right.$$

**Figure 5:**
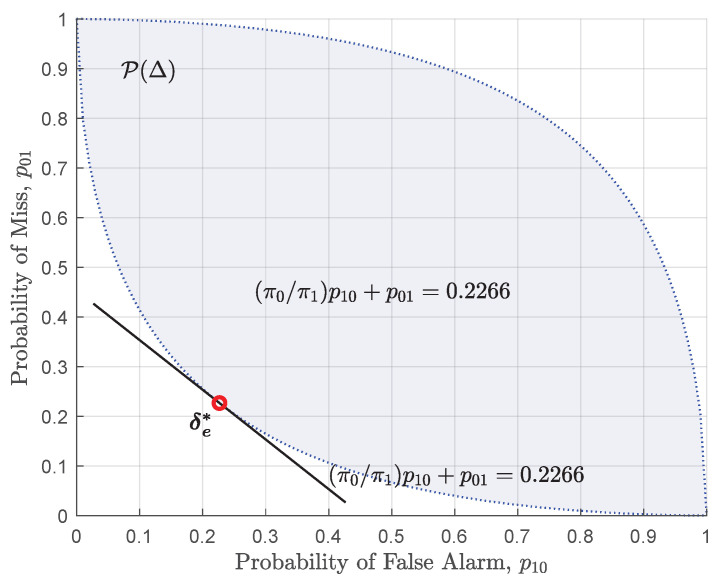
The optimal decision rule and the pairwise probability vector that minimizes the probability of error (Remark 2).

*It should be noted that the decision rule specified above is the MAP rule given in* (19)*. An illustrative example with the location of the pairwise probability vector of the decision rule given above, which results in a minimum probability of error value of 0.2266, is shown in Figure 5 with a red circle.*

NP Hypothesis Testing

In *NP hypothesis testing* (M=2), the objective is to maximize the detection probability $p_{11}$ while maintaining the false alarm probability $p_{10}$ below a specified level α. Since $p_{01}=1-p_{11}$, the NP criterion can equivalently be expressed as the following constrained optimization:

(60) $$\min_{\delta \in \Delta }\;p_{01}\;\mathrm{subject}\;\mathrm{to}\;p_{10}\leq \alpha \;.$$

Since the miss probability $p_{01}$ is a non-increasing function of the false alarm probability $p_{10}$, the optimal decision rule achieves the false alarm constraint with equality, $p_{10}=\alpha$, and takes the form of a randomized LRT. By definition, the NP rule achieves the minimum possible miss probability for a given false alarm level; geometrically, this implies that the pairwise probability vector of the NP rule resides on the lower boundary of P(Δ). According to Lemma 2, the optimal decision rule is a randomization among *at most two* deterministic decision rules, both associated with the same weight vector v.

The lower boundary of the set P(Δ) consists of pairwise probability vectors corresponding to standard NP decision rules. To characterize the upper boundary, we introduce the *flipped NP* criterion ([20] Lemma 2), which is provided in the lemma given below.

**Lemma** **3** ([20] Lemma 2 (Flipped Neyman–Pearson Rule))**.** *Let the observation vector y be distributed according to $f_{0}\left(y\right)$ and $f_{1}\left(y\right)$ under hypotheses $H_{0}$ and $H_{1}$, respectively. For any 0≤α≤1, let $\delta^{\mathrm{FNP}}$ be a decision rule c.f.* (22)*:*

(61) $$\delta_{1}^{\mathrm{FNP}}\left(y\right)=\left\{\begin{array}{cc} 1, & \mathrm{if}\;f_{1}\left(y\right)<\eta_{0}f_{0}\left(y\right)\\ \gamma_{0}, & \mathrm{if}\;f_{1}\left(y\right)=\eta_{0}f_{0}\left(y\right)\\ 0, & \mathrm{if}\;f_{1}\left(y\right)>\eta_{0}f_{0}\left(y\right) \end{array}\right.$$

*where $0\leq \gamma_{0}\leq 1$ and $\eta_{0}\geq 0$ are chosen such that the false alarm probability $p_{10}=\alpha$. Then, $\delta^{\mathrm{FNP}}$ maximizes the miss probability $p_{01}$ among all rules **δ** satisfying $p_{10}\geq \alpha$. That is, $\delta^{\mathrm{FNP}}$ solves:*

(62) $$\max_{\delta \in \Delta }\;p_{01}\;\mathrm{subject}\;\mathrm{to}\;p_{10}\geq \alpha \;.$$

Under the flipped NP criterion, the objective is to maximize the miss probability $p_{01}$ while maintaining the false alarm probability $p_{10}$ at a specific level α. Since the miss probability is a non-increasing function of the false alarm probability, the FNP rule achieves the constraint $p_{10}=\alpha$ with equality. By maximizing $p_{01}$ for a fixed $p_{10}$, the pairwise probability vectors of the FNP rules define the upper boundary of P(Δ). According to Lemma 2, the FNP rule can be implemented as a randomization between at most two deterministic decision rules sharing the same weight vector v. Hence, the flipped NP rule given in Lemma 3 and the characterization of the decision rule done by using Lemma 2 agree.

For a fixed false alarm probability α, the lower and upper boundaries of the set P(Δ) correspond to the minimum and maximum achievable miss probabilities, respectively. These extremes are achieved by the NP and flipped NP rules. Consequently, any arbitrary point $\tilde{p}={\left[{\tilde{p}}_{10},{\tilde{p}}_{01}\right]}^{⊤}\in P\left(\Delta \right)$ can be represented as a convex combination of the NP and flipped NP pairwise probability vectors, both evaluated at a false alarm probability $\alpha ={\tilde{p}}_{10}$:

(63) $$\tilde{p}=\lambda p^{\mathrm{NP}}\left(\delta^{\mathrm{NP}}\right)+\left(1-\lambda \right)p^{\mathrm{FNP}}\left(\delta^{\mathrm{FNP}}\right)\;,$$

where the randomization coefficient is given by $\lambda =\frac{{\tilde{p}}_{01}-p_{01}^{F}}{p_{01}^{N}-p_{01}^{F}}$. Thus, the decision rule $\tilde{\delta }$ achieving $\tilde{p}\left(\tilde{\delta }\right)$ written as the randomization of NP and flipped NP rules is given below:

(64) $$\tilde{\delta }=\lambda \delta^{\mathrm{NP}}+\left(1-\lambda \right)\delta^{\mathrm{FNP}}\;,$$

where the NP rule $\delta^{\mathrm{NP}}$ and the flipped NP rule $\delta^{\mathrm{FNP}}$ are employed with probabilities λ and 1−λ, respectively. For binary hypothesis testing, every point in P(Δ) is uniquely characterized by the false alarm probability α and the randomization constant λ. As a result, the entire set P(Δ) can be spanned by sweeping α∈[0,1] and λ∈[0,1].

## 4. Binary Hypothesis Testing with Prospect Theory-Based Behavioral Utility as Performance Metric

The rational performance metrics, such as Bayes risk and NP criterion, utilize the actual probability and utility or cost values; as a result, direct analysis of these performance metrics often reveals the structure of the optimal decision rules. Furthermore, the optimal pairwise probability vectors of rational performance metrics lie on the boundary of the P(Δ) set. In some hypothesis-testing problems, however, the DA or the designer of the decision rule may exhibit behavioral biases and therefore may not act as a fully rational agent, such as a physical sensor. In such cases, rational performance metrics may fail to capture the actual decision-making behavior of the DA. To model such behavior, PT-based approaches have been adopted in the literature [13,14,15,16,17,18,19,20,21], which employ a probability weighting function and a utility valuation function [22,23,24,25,26]. Since the probability weighting function introduces a nonlinear transformation of the objective probabilities, the resulting behavioral utility is generally nonlinear in the pairwise probability vector; therefore, unlike classical criteria, its optimizer need not lie on the boundary of P(Δ).

In this section, we focus on the problem of binary hypothesis testing with a DA whose performance metric is the PT-based behavioral utility that is computed using the perceived probability and utility values that are obtained using the probability weighting and utility valuation functions, which are specified in the following.

### 4.1. Prospect Theory (PT)

Originally developed by Daniel Kahneman and Amos Tversky in 1979, PT provides a robust framework for understanding how behavioral biases influence decision-making under uncertainty. In PT, it is posited that the behavioral biases of human DAs are driven by subjective perceptions of probability and utility. Specifically, unlike rational agents, such as physical sensors that operate on objective probability and utility values, human decision-makers perceive these quantities through subjective probability weighting and utility valuation functions [22,23,26,30].

**Figure 6:**
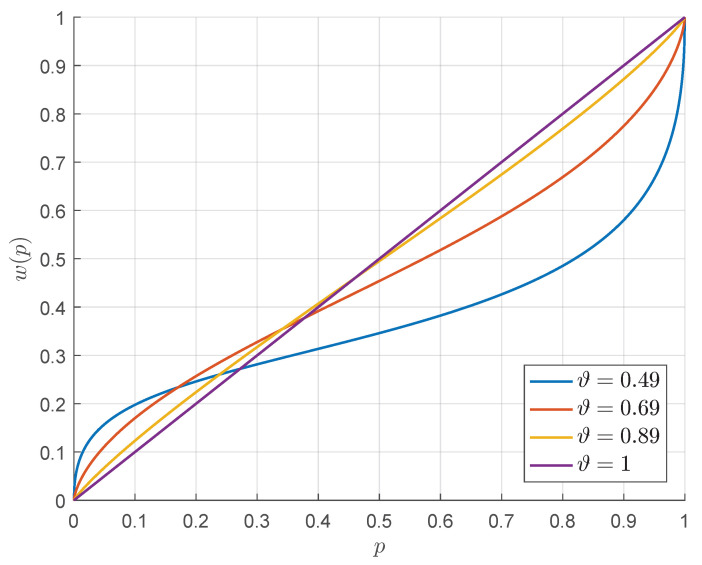
The probability weighting function in (65) for various values of ϑ∈{0.49,0.69,0.89,1}.

To model the human perception of probabilities, a probability weighting function, w(p), is employed in PT. A widely adopted formulation for this function is given by

(65) $$w\left(p\right)=\frac{p^{\vartheta }}{{\left(p^{\vartheta }+{\left(1-p\right)}^{\vartheta }\right)}^{1/\vartheta }}\;,$$

where p∈[0,1] represents the objective probability and w(p)∈[0,1] denotes its perceived version [31]. Here, ϑ serves as a tuning parameter, which satisfies ϑ>0.279 [25,32]. This function typically captures the human tendency to *overweigh low probabilities* and *underweigh high probabilities*. Examples are shown in Figure 6 for ϑ∈{0.49,0.69,0.89,1}.

**Figure 7:**
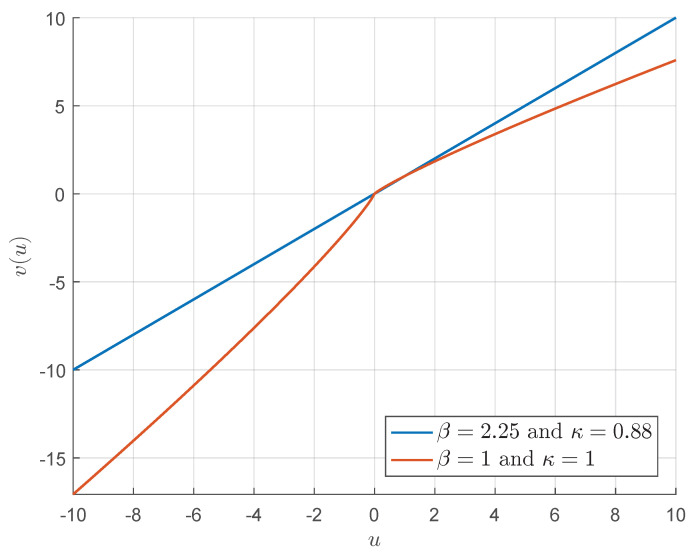
The utility valuation function with parameters $u_{r}=0$, β=2.25, and κ=0.88.

The subjective perception of an outcome’s utility is modeled by a utility valuation function, v(u), defined relative to a reference point $u_{r}$ as given below:

(66) $$v\left(u\right)=\left\{\begin{array}{cc} {\left(u-u_{r}\right)}^{\kappa }, & \mathrm{if}\;u\geq u_{r}\\ -\beta {\left(u_{r}-u\right)}^{\kappa }, & \mathrm{if}\;u<u_{r}\;, \end{array}\right.$$

where *u* represents the objective utility viewed as a gain if $u>u_{r}$ and a loss if $u<u_{r}$ [23]. Here, β>1 is the *loss-aversion* parameter, and κ∈(0,1) reflects *diminishing sensitivity*. The median behavioral parameters are estimated as β=2.25 and κ=0.88 [23,26]. The resulting utility valuation function, characterized by a steeper slope for losses than for gains, is depicted in Figure 7 for the standard case of $u_{r}=0$.

The median values for the behavioral parameters are estimated as ϑ=0.69, β=2.25, and κ=0.88 [23,26]. Furthermore, the behavioral parameters for a rational agent are ϑ=1, β=1, and κ=1. In Figure 6, the probability weighting function is depicted for various values of ϑ∈{0.49,0.69,0.89,1}. Similarly, the utility valuation function for a behavioral decision-maker with β=2.25, κ=0.88, and $u_{r}=0$ is provided in Figure 7.

### 4.2. Optimal Decision Rules for Behavioral Utility-Based Binary Hypothesis Testing

We assume that a behaviorally-biased DA, i.e., a human DA, is employed for binary hypothesis testing, where the conditional distributions of the observation vector Y are given by

(67) $$\begin{array}{cc} H_{0} & :Y∼f_{0} \end{array}$$

(68) $$\begin{array}{cc} H_{1} & :Y∼f_{1} \end{array}$$

The DA evaluates the performance using a behavioral utility metric, expressed as

(69) $$U\left(p\left(\delta \right)\right)=\sum_{j=0}^{1}\sum_{i=0}^{1}w\left(p_{ij}\pi_{j}\right)v\left(u_{ij}\right)$$

where $\pi_{j}$ is the prior probability of hypothesis $H_{j}$, and $u_{ij}$ denotes the actual utility associated with choosing $H_{i}$ when $H_{j}$ is true, which can be considered as the negative of the cost value, $c_{ij}$, in (6). Here, *w* and *v* represent the probability weighting and utility valuation functions, respectively (see (65) and (66)).

The behavioral utility performance metric in (69) utilizes perceived probability and utility values rather than their objective counterparts. Consequently, it can be viewed as a behavioral variant of the Bayes risk (with a reverse sign, as it is defined as utility). The fundamental differences are: (i) instead of absolute costs, the metric employs utility values $u_{ij}$, where negative values represent perceived losses and positive values represent perceived gains, and (ii) unlike a rational agent who utilizes actual probability and cost values, the behavioral agent processes these through non-linear functions *w* and *v*, capturing human-like cognitive biases.

For this problem, the optimal decision rule maximizes the behavioral utility and is obtained by solving the following optimization problem:

(70) $$\delta^{*}=\underset{\delta \in \Delta }{\arg\;\max}\;U\left(p\left(\delta \right)\right)\;.$$

Expanding the behavioral utility in (69) in terms of the pairwise error probabilities for the binary case ($p_{00}=1-p_{10}$ and $p_{11}=1-p_{01}$), we obtain

(71) $$\begin{array}{cc} U\left(p\left(\delta \right)\right)=w\left(\pi_{0}\left(1-p_{10}\right)\right)v\left(u_{00}\right)+ & w(\pi_{0}p_{10})v\left(u_{10}\right)\\ & \;\;\;\;+w\left(\pi_{1}p_{01}\right)v\left(u_{01}\right)+w\left(\pi_{1}\left(1-p_{01}\right)\right)v\left(u_{11}\right)\;. \end{array}$$

A decision rule, δ, influences the behavioral utility solely through its pairwise probability vector, p(δ). Consequently, maximizing the behavioral utility over the set of all randomized decision rules, Δ, is equivalent to maximizing the function U(p) over the set of all achievable pairwise probability vectors, P(Δ):

(72) $$p^{*}\left(\delta^{*}\right)=\underset{p\in P(\Delta )}{\arg\;\max}\;U\left(p\right)\;.$$

The optimization of behavioral utility is thus a specific instance of the general problem formulated in (25). Therefore, the optimal decision rules can be characterized using Theorem 1. Notably, as detailed in ([16] Proposition 1), certain utility configurations can result in scenarios where a standard LRT is no longer the optimal strategy. ([16] Proposition 1) is summarized in the following proposition.

**Proposition** **1** ([16] Proposition 1)**.** *Suppose that the probability weighting function w is monotonically increasing.*

- *Case (a): If $v\left(u_{10}\right)v\left(u_{00}\right)<0$ or $v\left(u_{11}\right)v\left(u_{01}\right)<0$, then the solution to* (72) *is a randomized LRT. This rule takes the form of either a standard NP rule, $\delta^{\mathrm{NP}}$, or a flipped NP rule, $\delta^{\mathrm{FNP}}$:*
  (73) $$\begin{array}{cc} \delta_{1}^{\mathrm{NP}}\left(y\right) & =\left\{\begin{array}{cc} 1, & \mathrm{if}\;L\left(y\right)>\eta_{1}\\ \gamma_{1}, & \mathrm{if}\;L\left(y\right)=\eta_{1}\\ 0, & \mathrm{if}\;L\left(y\right)<\eta_{1} \end{array}\right. \end{array}$$
  (74) $$\begin{array}{cc} \delta_{1}^{\mathrm{FNP}}\left(y\right) & =\left\{\begin{array}{cc} 1, & \mathrm{if}\;L\left(y\right)<\eta_{2}\\ \gamma_{2}, & \mathrm{if}\;L\left(y\right)=\eta_{2}\\ 0, & \mathrm{if}\;L\left(y\right)>\eta_{2} \end{array}\right. \end{array}$$
- *Case (b): If $v\left(u_{10}\right)v\left(u_{00}\right)\geq 0$ and $v\left(u_{11}\right)v\left(u_{01}\right)\geq 0$, then the optimal decision rule may require a randomization of multiple LRTs.*

Unlike Bayesian risk, which utilizes objective probability and cost values, the optimality of a single LRT cannot be guaranteed in behavioral utility-based binary hypothesis testing. This is primarily due to the non-linearity of the probability weighting function, which overweights low probabilities and underweights high probabilities. Consequently, the position of the optimal pairwise probability vector $p^{*}\left(\delta^{*}\right)$ within the achievable set P(Δ) depends heavily on the signs of the utility valuation functions. When the conditions for Case (a) are met, i.e., $v\left(u_{10}\right)v\left(u_{00}\right)<0$ or $v\left(u_{11}\right)v\left(u_{01}\right)<0$ holds, the optimal pairwise probability vector $p^{*}\left(\delta^{*}\right)$ resides on the boundary of P(Δ). In this scenario, Lemma 2 applies directly, and the optimal rule is a randomization among at most two deterministic decision rules constructed with the same weight vector v.

Conversely, if the conditions $v\left(u_{10}\right)v\left(u_{00}\right)\geq 0$ and $v\left(u_{11}\right)v\left(u_{01}\right)\geq 0$ holds, i.e., Case (b) is valid, the optimal vector $p^{*}\left(\delta^{*}\right)$ *may* reside in the interior of the P(Δ) set. Under these circumstances, the structure of the optimal rule depends on the measure of the boundary set B(v). If the boundary points of y occur with a nonzero probability measure, the optimal decision rule is a randomization between two possibly randomized LRTs with distinct weight vectors. More explicitly, one of these LRTs can always be chosen as a deterministic LRT; however, the other LRT may be a randomized LRT, which randomizes two LRTs with the same weight vector. Since the optimization problem given in (72) is a special case of the general framework given in (25), by leveraging Theorem 1, the following corollary can be devised to characterize the optimal decision rules for behavioral utility.

**Corollary** **1.** *For PT-based binary hypothesis testing, the optimal decision rule can be expressed as a randomization among at most three deterministic decision rules based on the likelihood ratio $L\left(y\right)=f_{1}\left(y\right)/f_{0}\left(y\right)$. The first decision rule can always be chosen as*

(75) $$\delta^{1}\left(y\right)=\left\{\begin{array}{cc} 1, & L\left(y\right)>\eta_{1}\\ 0\;\mathrm{or}\;1, & L\left(y\right)=\eta_{1}\\ 0, & L\left(y\right)<\eta_{1} \end{array}\right.\;,$$

*and the other two decision rules form either an NP rule or a flipped NP rule. If they form an NP rule, they are characterized as*

(76a) $$\delta^{2}\left(y\right)=\left\{\begin{array}{cc} 1, & L\left(y\right)\geq \eta_{2}\\ 0, & L\left(y\right)<\eta_{2} \end{array}\right.$$

(76b) $$\delta^{3}\left(y\right)=\left\{\begin{array}{cc} 1, & L\left(y\right)>\eta_{2}\\ 0, & L\left(y\right)\leq \eta_{2} \end{array}\right.$$

*Conversely, if the other two decision rules form a flipped NP rule, they are characterized as*

(77a) $$\delta^{2}\left(y\right)=\left\{\begin{array}{cc} 1, & L\left(y\right)\leq \eta_{3}\\ 0, & L\left(y\right)>\eta_{3} \end{array}\right.$$

(77b) $$\delta^{3}\left(y\right)=\left\{\begin{array}{cc} 1, & L\left(y\right)<\eta_{3}\\ 0, & L\left(y\right)\geq \eta_{3} \end{array}\right.$$

This corollary is a direct application of Theorem 1 to the behavioral utility. Geometrically, let $\delta^{1}$ be a deterministic decision rule associated with a weight vector $v^{1}$. Due to its structure, its pairwise probability vector resides on the lower boundary of P(Δ). A ray originating from its pairwise probability vector $p^{1}\left(\delta^{1}\right)$ intersects the boundary of the set P(Δ) at a point that is not necessarily an extreme point of the set. If this intersection point is located on the lower boundary of P(Δ), it can be achieved via an NP rule; thus, a randomization of the decision rules in (76) yields the intersection point. Conversely, if the intersection point is located on the upper boundary of P(Δ), the randomization of the decision rules in (77) is required, i.e., the intersection point corresponds to a pairwise probability vector of a flipped NP rule.

If the observation Y is governed by a continuous distribution such that the boundary points occur with zero probability measure, every point on the boundary of P(Δ) becomes an extreme point. In such cases, the optimal decision rule simplifies to a randomization of at most two deterministic LRTs with different weight vectors. Moreover, if Case 1 of Proposition 1 holds, the optimal decision rule is either a deterministic LRT in the case where the boundary points of the Y occur with zero probability or a randomized LRT that can be written as a randomization of two deterministic LRTs with the same weight vector, v, in the case the boundary points of Y occurring with non-zero probabilities.

An alternative approach for characterizing the optimal decision rule that maximizes behavioral utility is presented in ([16] Proposition 2) and also in ([20] Proposition 1), which are provided in the proposition given below. Based on the geometric structure of the achievable set, the boundary of P(Δ) consists of points corresponding to either NP rules or flipped NP rules. Consequently, the optimal pairwise probability vector $p^{*}$ must be achieved by one of the following: (i) a standard NP rule, (ii) a flipped NP rule, or (iii) a randomization between an NP rule and a flipped NP rule that share the same false alarm probability α. This third case effectively allows for the exploration of the interior of P(Δ) by taking convex combinations of the lower and upper boundaries at any given false-alarm level.

**Proposition** **2** ([16] Proposition 2)**.** *Let $p^{*}\left(\delta^{*}\right)={\left[p_{10}^{*},p_{01}^{*}\right]}^{⊤}$ be the optimal pairwise probability vector that solves the behavioral utility maximization problem in* (72)*. The corresponding optimal decision rule $\delta^{*}$ can be implemented as a randomized rule:*

(78) $$\delta^{*}=\lambda \delta^{\mathrm{NP}}+\left(1-\lambda \right)\delta^{\mathrm{FNP}}\;,$$

*where $\delta^{\mathrm{NP}}$ and $\delta^{\mathrm{FNP}}$ are the NP and flipped NP rules, respectively, both designed with size $\alpha =p_{10}^{*}$. Let $p_{01}^{N,*}$ and $p_{01}^{F,*}$ denote the miss probabilities associated with $\delta^{\mathrm{NP}}$ and $\delta^{\mathrm{FNP}}$ at this false alarm level. The randomization coefficient is then given by*

(79) $$\lambda =\frac{p_{01}^{F,*}-p_{01}^{*}}{p_{01}^{F,*}-p_{01}^{N,*}}\;\cdot$$

*Specifically, the optimal rule $\delta^{*}$ selects the decision outcome of $\delta^{\mathrm{NP}}$ with probability λ and the outcome of $\delta^{\mathrm{FNP}}$ with probability 1−λ.*

The fundamental intuition behind Proposition 2 is that any point $\tilde{p}={\left[{\tilde{p}}_{10},{\tilde{p}}_{01}\right]}^{⊤}$ within the achievable set P(Δ) can be realized through a specific randomized decision rule:

(80) $$\tilde{\delta }\left(y\right)=\lambda \delta^{\mathrm{NP}}+\left(1-\lambda \right)\delta^{\mathrm{FNP}}\;,$$

where $\delta^{\mathrm{NP}}$ and $\delta^{\mathrm{FNP}}$ are the NP and flipped NP decision rules of size $\alpha ={\tilde{p}}_{10}$, respectively. In this framework, the randomization constant λ dictates the probability with which the NP rule is selected over the flipped NP rule. Geometrically, the lower and upper boundaries of the set P(Δ) are defined by the NP and flipped NP rules across all possible sizes. Consequently, if the optimal pairwise probability vector $p^{*}\left(\delta^{*}\right)$ lies on the lower boundary, we set λ=1 to recover the standard NP rule of size ${\tilde{p}}_{10}$. Conversely, if $p^{*}\left(\delta^{*}\right)$ resides on the upper boundary, the randomization constant is set to λ=0 to employ the flipped NP rule. For the case where $p^{*}\left(\delta^{*}\right)$ is located in the interior of P(Δ), the randomization of these two boundary rules with $\lambda =\left(p_{01}^{F,*}-p_{01}^{*}\right)/\left(p_{01}^{F,*}-p_{01}^{N,*}\right)$ ensures that the desired miss probability is achieved at the target false alarm level.

The approach given in Proposition 2 effectively "scans" the vertical interior of the achievable set, providing a robust implementation of optimal rules. Furthermore, using Proposition 2, it is possible to devise a practical algorithm to obtain the decision rule that maximizes the behavioral utility, which consists of the following steps:

1. **Solve for $p^{*}\left(\delta^{*}\right)$:** Solve the optimization problem in (72) to obtain the optimal pairwise probability vector $p^{*}\left(\delta^{*}\right)={\left[p_{10}^{*},p_{01}^{*}\right]}^{⊤}$.
2. **Design Boundary Rules:** Design the standard NP and flipped NP rules as LRTs defined in (73) and (74), both set to a false alarm probability of $\alpha =p_{10}^{*}$.
3. **Randomize:** Combine these two rules using the randomization constant λ derived in Proposition 2 to achieve the target miss probability $p_{01}^{*}$.

## 5. Numerical Examples

In this section, we provide several numerical examples to corroborate the theoretical results established in the previous sections based on Theorem 1. To this end, we consider a behaviorally biased DA employed to detect the true state of a POI. The POI resides in one of two states, denoted by hypotheses $H_{0}$ and $H_{1}$, which occur with prior probabilities $\pi_{0}=\pi_{1}=0.5$. The performance of the behavioral DA is evaluated using the behavioral utility function defined in (69).

In the first example, we assume that the DA receives binary reports from two sources; specifically, these sources send their local observations regarding the actual state of the POI to the DA. Consequently, the set of observation vectors available to the DA is $Y=\{{\left[0,0\right]}^{⊤},{\left[0,1\right]}^{⊤},{\left[1,0\right]}^{⊤},{\left[1,1\right]}^{⊤}\}$. The conditional probability mass functions (PMFs) under $H_{0}$ and $H_{1}$ are provided in Table 1. The perceived utilities and the probability weighting parameter of the behavioral DA are set to $v(u_{00})=1.1$, $v(u_{01})=0.6$, $v(u_{10})=0.5$, $v(u_{11})=1$, and ϑ=0.45 (see (65) and (69)).

**Figure 8:**
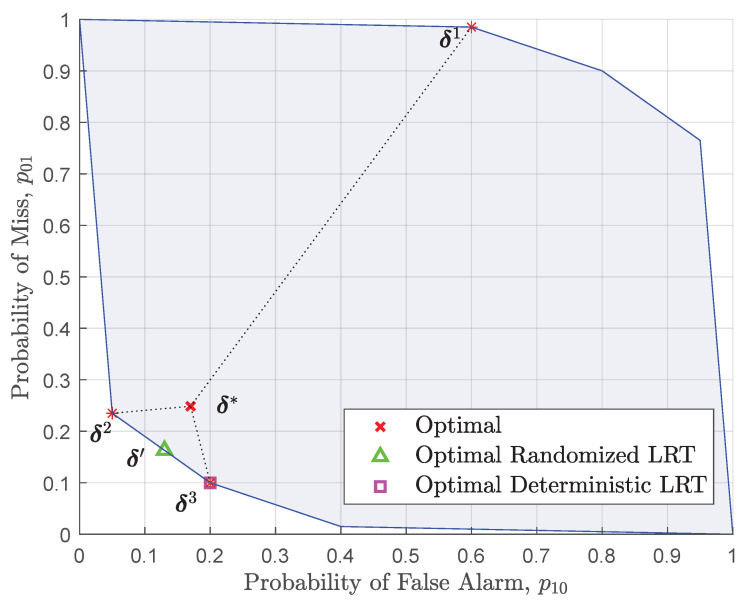
Optimal pairwise probability vectors maximizing the behavioral utility for a case where the optimal solution resides in the *interior* of the feasible set P(Δ) shown by the shaded area.

In Figure 8, we highlight the boundary of P(Δ) with solid blue lines. The maximum value of the behavioral utility U=0.8181 is achieved with a pairwise probability vector of $p^{*}\left(\delta^{*}\right)={\left[0.17,0.2486\right]}^{⊤}$, which is shown with a red x sign. This pairwise probability vector can be obtained by using a randomized decision rule that is given below:

(81) $$\delta^{*}=\lambda^{1}\delta^{1}+\lambda^{2}\delta^{2}+\lambda^{3}\delta^{3}\;,$$

where $\lambda^{1}=0.0977$, $\lambda^{2}=0.4605$, and $\lambda^{3}=0.4418$, and the decision rules $\delta^{1}$, $\delta^{2}$ and $\delta^{3}$ are specified as follows:

(82a) $$\delta_{1}^{1}=\left\{\begin{array}{cc} 1 & \;\mathrm{if}\;L(y)<0.425\\ 0 & \;\mathrm{if}\;L(y)\geq 0.425 \end{array}\right.$$

(82b) $$\delta_{1}^{2}=\left\{\begin{array}{cc} 1 & \;\mathrm{if}\;L(y)>0.9\\ 0 & \;\mathrm{if}\;L(y)\leq 0.9 \end{array}\right.$$

(82c) $$\delta_{1}^{3}=\left\{\begin{array}{cc} 1 & \;\mathrm{if}\;L(y)\geq 0.9\\ 0 & \;\mathrm{if}\;L(y)<0.9\;. \end{array}\right.$$

Since $\delta^{2}$ and $\delta^{3}$ are constructed with the same weight vector, the optimal decision rule given in (81) can be written as

(83) $$\delta^{*}=\lambda^{1}\delta^{1}+\lambda^{23}\delta^{23}\;,$$

where $\lambda^{1}=0.0977$, $\lambda^{23}=0.9023$, and $\delta^{23}$ is

(84) $$\delta_{1}^{23}=\left\{\begin{array}{cc} 1 & \;\mathrm{if}\;L(y)>0.9\\ 0.5103 & \;\mathrm{if}\;L(y)=0.9\\ 0 & \;\mathrm{if}\;L(y)<0.9\;, \end{array}\right.$$

which randomly selects $\delta^{2}$ or $\delta^{3}$ with probabilities 0.5103 and 0.4897, respectively. The pairwise probability vectors achieved by the decision rules specified above are marked in Figure 8.

Alternatively, the decision rules given above can be expressed as

(85a) $$\delta_{1}^{1}=\left\{\begin{array}{cc} 1 & \;\mathrm{if}\;y={\left[0,0\right]}^{⊤}\\ 0 & \;\mathrm{otherwise}\; \end{array}\right.$$

(85b) $$\delta_{1}^{23}=\left\{\begin{array}{cc} 1 & \;\mathrm{if}\;y={\left[1,1\right]}^{⊤}\\ 0.5103 & \;\mathrm{if}\;y={\left[0,1\right]}^{⊤}\\ 0 & \;\mathrm{if}\;y\in \{{\left[0,0\right]}^{⊤},{\left[1,0\right]}^{⊤}\}\;. \end{array}\right.$$

where $\delta^{23}$ can be written as a randomization $\delta^{2}$ and $\delta^{3}$, which are given below:

(86a) $$\delta_{1}^{2}=\left\{\begin{array}{cc} 1 & \;\mathrm{if}\;y={\left[1,1\right]}^{⊤}\\ 0 & \;\mathrm{otherwise}\; \end{array}\right.$$

(86b) $$\delta_{1}^{3}=\left\{\begin{array}{cc} 1 & \;\mathrm{if}\;y\in \{{\left[0,1\right]}^{⊤},{\left[1,1\right]}^{⊤}\}\\ 0 & \;\mathrm{if}\;y\in \{{\left[0,0\right]}^{⊤},{\left[1,0\right]}^{⊤}\}\;. \end{array}\right.$$

Note that $y={\left[0,1\right]}^{⊤}$ is a boundary point of y for $v^{23}={\left[1,\;0.9\right]}^{⊤}$.

For comparison, we assume a scenario where the DA is restricted to employing either a randomized LRT (i.e., an NP or flipped NP rule) or a deterministic decision rule as defined in (11). In this case, the achievable pairwise probability vectors are limited to the boundary and the extreme points of the set P(Δ), respectively. When a randomized decision rule is employed, the maximum behavioral utility is U=0.8155, achieved at the pairwise probability vector $p\left(\delta \right)={\left[0.13,0.163\right]}^{⊤}$. This point is represented by a green triangle in Figure 8 and corresponds to the decision rule $\delta^{\prime }$ specified by

(87) $$\delta_{1}^{\prime }\left(y\right)=\left\{\begin{array}{cc} 1, & \mathrm{if}\;L(y)>0.9\\ 0.5333, & \mathrm{if}\;L(y)=0.9\\ 0, & \mathrm{if}\;L(y)<0.9 \end{array}\right.\;,$$

which is implemented by randomizing between the deterministic decision rules $\delta^{2}$ in (82b) and $\delta^{3}$ in (82c) with probabilities 0.5333 and 0.4667, respectively. Furthermore, if the DA is restricted solely to deterministic LRTs, the maximum behavioral utility drops to U=0.8105, which is achieved by the decision rule $\delta^{3}$ defined in (82c). The corresponding pairwise probability vector is indicated by a purple square in Figure 8.

**Figure 9:**
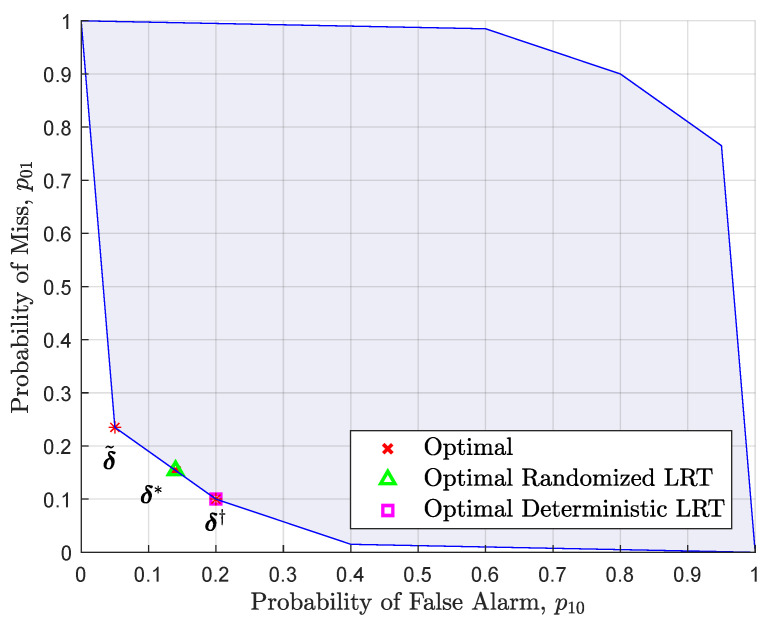
Optimal pairwise probability vectors maximizing behavioral utility for a case where the optimal solution lies on the boundary of P(Δ).

In the second example, we set the DA’s probability weighting parameter to ϑ=0.69 in (65), and the perceived utility values of the DA are $v(u_{00})=1.3$, $v(u_{01})=0.4$, $v(u_{10})=0.4$, and $v(u_{11})=1.2$. The optimal pairwise probability vector that yields the maximum behavioral utility value, U=1.1341, is located on the boundary of the P(Δ) set. Furthermore, since the boundary values of Y occur with non-zero probability, Lemma 2, Case (b) holds, and the optimal decision rule is a randomization of at most two deterministic decision rules. The optimal pairwise probability vector $p^{*}\left(\delta^{*}\right)={\left[0.11,0.181\right]}^{⊤}$ is indicated by a red ‘x’ in Figure 9, corresponding to the following decision rule:

(88) $$\delta_{1}^{*}\left(y\right)=\left\{\begin{array}{cc} 1, & \mathrm{if}\;L(y)>0.9\\ 0.4, & \mathrm{if}\;L(y)=0.9\\ 0, & \mathrm{if}\;L(y)<0.9\;, \end{array}\right.$$

which employs the deterministic decision rules given below with probabilities 0.4 and 0.6, respectively:

(89a) $${\tilde{\delta }}_{1}\left(y\right)=\left\{\begin{array}{cc} 1, & \mathrm{if}\;L(y)\geq 0.9\\ 0, & \mathrm{if}\;L(y)<0.9 \end{array}\right.$$

(89b) $$\delta_{1}^{†}\left(y\right)=\left\{\begin{array}{cc} 1, & \mathrm{if}\;L(y)>0.9\\ 0, & \mathrm{if}\;L(y)\leq 0.9\;. \end{array}\right.$$

If the DA is restricted to purely deterministic decision rules, the maximum behavioral utility value drops to U=1.1304, achieved by the deterministic rule $\tilde{\delta }$ defined in (89a). The pairwise probability vector corresponding to $\tilde{\delta }$ is $\tilde{p}\left(\tilde{\delta }\right)={\left[0.05,0.235\right]}^{⊤}$, indicated in Figure 9 by a purple square.

**Figure 10:**
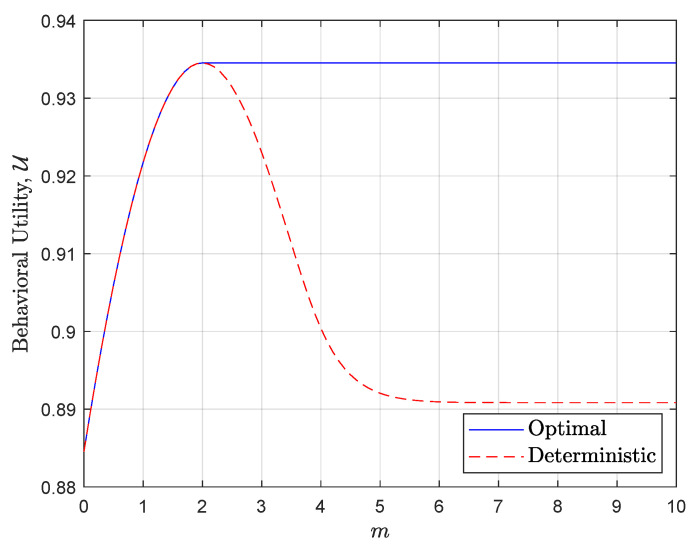
Maximum behavioral utility as a function of the parameter m∈[0,10], comparing the performance achieved by the optimal (potentially randomized) rules against purely deterministic decision rules.

In the third example, we assume that the observation of the DA has a continuous distribution under both hypotheses, and the conditional pdfs of observation are given below:

(90) $$\begin{array}{cc} H_{0}\; & :\;Y∼N(0,1) \end{array}$$

(91) $$\begin{array}{cc} H_{1}\; & :\;Y∼N(m,1)\;, \end{array}$$

where *m* is the mean value of observations under $H_{1}$. The probability weighting parameter of the DA is set to ϑ=0.5 in (65), and the perceived utilities are $v(u_{00})=1.2$, $v(u_{01})=0.4$, $v(u_{10})=0.6$, $v(u_{11})=1.1$ in (69). The maximum behavioral utility value that can be achieved with a decision rule as characterized in Theorem 1 and a deterministic decision rule as a function of $m\in \left[0,10\right]$ is given in Figure 10 with blue solid and red dashed lines, respectively. For m≤2, the maximum behavioral utility that is achieved with decision rules characterized using Theorem 1 and deterministic decision rules is the same. For m>2, the maximum behavioral utility that can be achieved with deterministic decision rules begins to drop while the maximum behavioral utility that can be achieved using the decision rules characterized in Theorem 1 stays the same.

**Figure 11:**
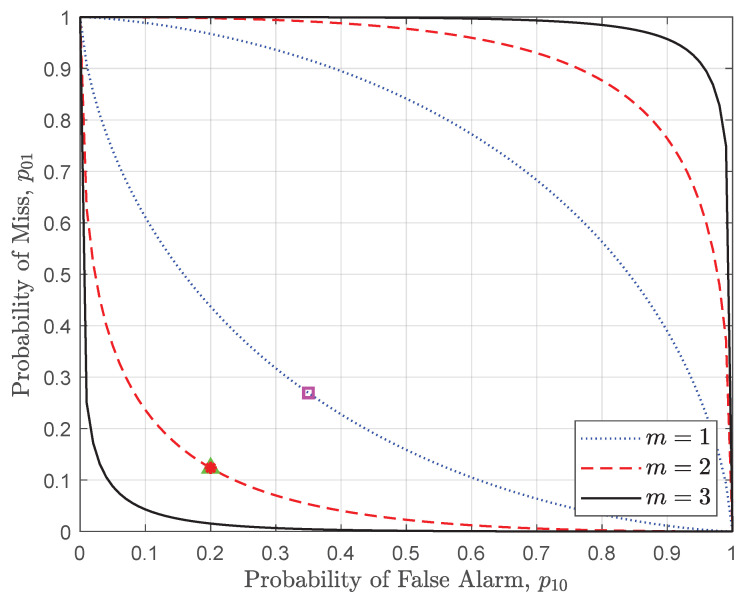
The feasible set of all achievable pairwise probability vectors P(Δ), highlighting the optimal vectors attained through randomized rules for specific values of m∈{1,2,3}.

To elucidate the performance gap between deterministic and randomized decision rules, we depict the set P(Δ) for m=1, m=2, and m=3 in Figure 11. Deterministic decision rules can only yield pairwise probability vectors that lie on the boundary of the P(Δ) set; the deterministic vectors yielding the maximum behavioral utility for m=1,2,3 are indicated by a magenta square, a green triangle, and a red star, respectively.

For m≤2, the optimal pairwise probability vector is located on the boundary of the P(Δ) set; thus, deterministic rules and the rules characterized via Theorem 1 achieve identical behavioral utility values. However, for m>2, the optimal vector no longer resides on the boundary, rendering it unachievable via deterministic rules. As *m* increases, the P(Δ) set expands, causing the distance between the optimal interior vector and the achievable boundary vectors to grow. This results in the observed decline in the maximum utility achieved by deterministic rules. Conversely, the characterization in Theorem 1 enables the selection of pairwise probability vectors in the interior of P(Δ) through randomization. Consequently, the expansion of the achievable set with increasing *m* does not negatively impact the maximum behavioral utility achieved when using the proposed optimal randomized rules.

## 6. Conclusions

In this paper, we present a comprehensive review of the general framework to characterize the optimal decision rules for *M*-ary hypothesis testing, where the performance metric is defined as a function of the pairwise (error) probabilities. This framework was originally developed in our previous work. The pairwise probabilities represent the probability of selecting a specific hypothesis given that a different hypothesis is true. These pairwise error probabilities are collected into a single vector, referred to as the pairwise probability vector. Then, to identify the optimal decision rules, an indirect approach is adopted, which focuses on the compact and convex set of all achievable pairwise probability vectors, P(Δ), instead of optimizing a given performance metric over the infinite-dimensional set of all possible randomized decision rules. By leveraging the topological properties of P(Δ), we have presented a theorem that characterizes the structure of the optimal decision rules based on the specific location of the optimal pairwise probability vector. It is established that any achievable pairwise probability vector can be realized through a randomization of at most two possibly randomized LRQs, each constructed with a specific set of parameters.

For *M*-ary hypothesis testing, an LRQ partitions the likelihood ratio space into *M* decision regions, which are defined as convex polytopes. The distinction between deterministic and randomized LRQs arises when an observation falls on a hyperplane that separates polytopes corresponding to different hypotheses; in such cases, a deterministic LRQ selects one hypothesis uniquely, whereas a randomized LRQ employs randomization. Furthermore, we have demonstrated that any randomized LRQ can be expressed as a randomization of at most M(M−1) deterministic LRQs that utilize the same parameter sets and differ only in their tie-breaking rules on the separating hyperplanes.

Several special cases have been highlighted in this work. First, if an optimal pairwise probability vector resides on the boundary of the feasible set, a single LRQ (either deterministic or randomized) is sufficient to achieve that performance. Second, if the likelihood ratio vector is continuously distributed under all hypotheses, any optimal pairwise probability vector in the interior of the feasible set can be achieved via a randomization of two deterministic LRQs corresponding to two distinct parameter sets. Finally, if the optimal pairwise probability vector is an extreme point of the feasible set, a single deterministic LRQ constitutes the optimal decision rule.

Moreover, we have evaluated a special case in which DA’s performance is measured using a PT-based behavioral utility function. This metric incorporates perceived probabilities and utilities, which are derived by applying non-linear probability weighting and utility valuation functions to the objective probability and cost values, respectively. Due to the inherent non-linearity of the probability weighting function, direct analytical approaches often fail to reveal the optimal decision rules. Furthermore, it is established that LRTs, which are optimal for rational performance metrics such as Bayes risk and the NP criterion, may be suboptimal in a behavioral context. In these instances, the optimal pairwise probability vector typically resides in the interior of the feasible set. As the pairwise probability vectors of standard LRTs are restricted to the boundary of the feasible set, they yield suboptimal results for behavioral agents. However, this study establishes that the randomized decision rules characterized by the presented theorem can successfully achieve these interior pairwise probability vectors, thereby optimizing performance in non-linear behavioral frameworks. We have corroborated the theoretical results with numerical examples that consider both discrete and continuous observation distributions. In all evaluated scenarios, the performance of the DA has been measured using the behavioral utility function. These results demonstrate that the proposed method for characterizing optimal decision rules yields performance metrics that are either identical or superior to those achieved by standard LRTs.

In summary, by consolidating these diverse scenarios into a single theory that leverages the geometry of the set of achievable pairwise probability vectors, this work bridges the gap between classical rational models, such as Bayes risk and NP criterion, and complex behavioral frameworks. The flexibility of this representation ensures that, whether an agent is operating under Bayesian risk or PT-based distortions, the optimal strategy can be identified and implemented through a consistent, finite-dimensional optimization. A possible avenue for future research is to focus on extending this unified representation to sequential hypothesis testing, where the geometry of the feasible set evolves dynamically over time.

## Figures and Tables

**Table 1:** The conditional probability mass functions of Y under $H_{0}$ and $H_{1}$.

True Hypothesis	*y* = [0, 0]^⊤^	*y* = [0, 1]^⊤^	*y* = [1, 0]^⊤^	*y* = [1, 1]^⊤^
$H_{0}$	0.6	0.15	0.2	0.05
$H_{1}$	0.015	0.135	0.085	0.765

## Data Availability

Data sharing is not applicable.
